# Chemical Composition, Biological Activities and Application of *Lupinus angustifolius* in Cosmetics and Food Industry

**DOI:** 10.3390/molecules31162918

**Published:** 2026-08-20

**Authors:** Maciej Jakobina, Renata Galek, Marta Preisner

**Affiliations:** Department of Plant Breeding and Seed Production, Wrocław University of Environmental and Life Sciences, Grunwaldzki Square 24a, 50-363 Wrocław, Poland; renata.galek@upwr.edu.pl (R.G.); marta.preisner@upwr.edu.pl (M.P.)

**Keywords:** *Lupinus angustifolius*, lupin, chemical composition, medicine, cosmetic, food, β-conglutin, lutein

## Abstract

Lupins have been cultivated since ancient times. Poland ranks second in the world in terms of lupin cultivation area (second only to Australia), particularly for narrow-leaved lupin. It is used in various industries. The purpose of this review is to analyze data on the chemical composition of narrow-leaved lupin in comparison with other species of this genus, as well as to identify its potential applications. The literature data characterize narrow-leaved lupin in terms of its content of protein, lectins, fat, carotenoids, phytosterols, fiber, sugars, alkaloids, vitamins, flavonoids, phenolic compounds, volatile organic compounds, and micro- and macronutrients. Due to its interesting qualitative and quantitative composition, this species may play a significant role in human nutrition—not only as an alternative source of protein, but above all as a source of health-promoting compounds. Studies conducted on humans and animals have demonstrated a wide range of biological effects, including anti-inflammatory, antioxidant and cholesterol-lowering effects. In addition, in vitro studies suggest anticancer effects. Among the tangible outcomes of research and commercialization efforts is the availability on the consumer market of food and cosmetic products containing lupin. Due to its properties, this species has a wide range of applications. However, to meet the requirements of various industrial sectors, a collaborative approach is necessary among plant breeders, scientists, and industry representatives working to improve this species. Only through joint efforts can the use of narrow-leaved lupin be expanded.

## 1. Introduction

**Figure 1:**
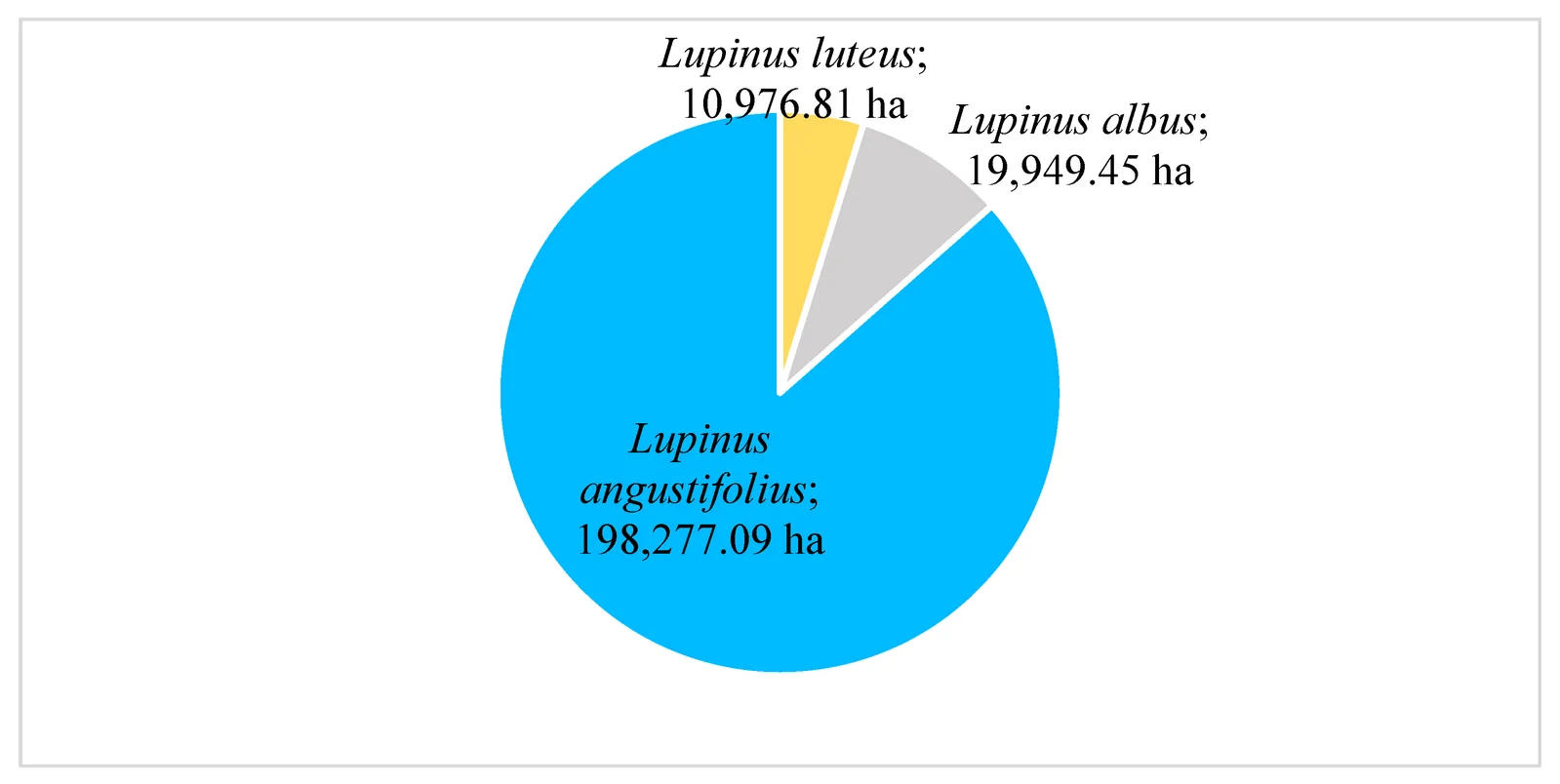
Area of lupin cultivation in Poland in 2025 [14].

Species of the genus *Lupinus* have been cultivated since ancient times, primarily as green manure and as a crop in crop rotation systems used for human and animal nutrition [1,2,3]. Advancement in lupin breeding—resulting in the acquisition of plants producing non-cracking pods and soft seeds of reduced alkaloid content—has contributed to the selection of forms suitable for large-scale cultivation [4]. Sweet varieties are currently available with low alkaloid content not exceeding 0.02 or 0.01%, as well as bitter varieties containing higher levels of alkaloids [5]. Currently, breeding is focused on the creation of “bitter/sweet” cultivars [6], those whose seeds do not contain large amounts of alkaloids, whilst other parts of the plant contain greater quantities, which would be immune to diseases caused by viruses [7] and pathogenic fungi [8], and also early cultivars [9], thermoneutral ones [10] and those resistant to drought [11]. The narrow-leaved lupin, originating from the Mediterranean Sea region [12,13], belongs to the most frequently grown crop species from the genus *Lupinus* both in Poland and worldwide, whose cultivation area equaled 229,206.35 ha in 2025 [14] (Figure 1).

**Figure 2:**
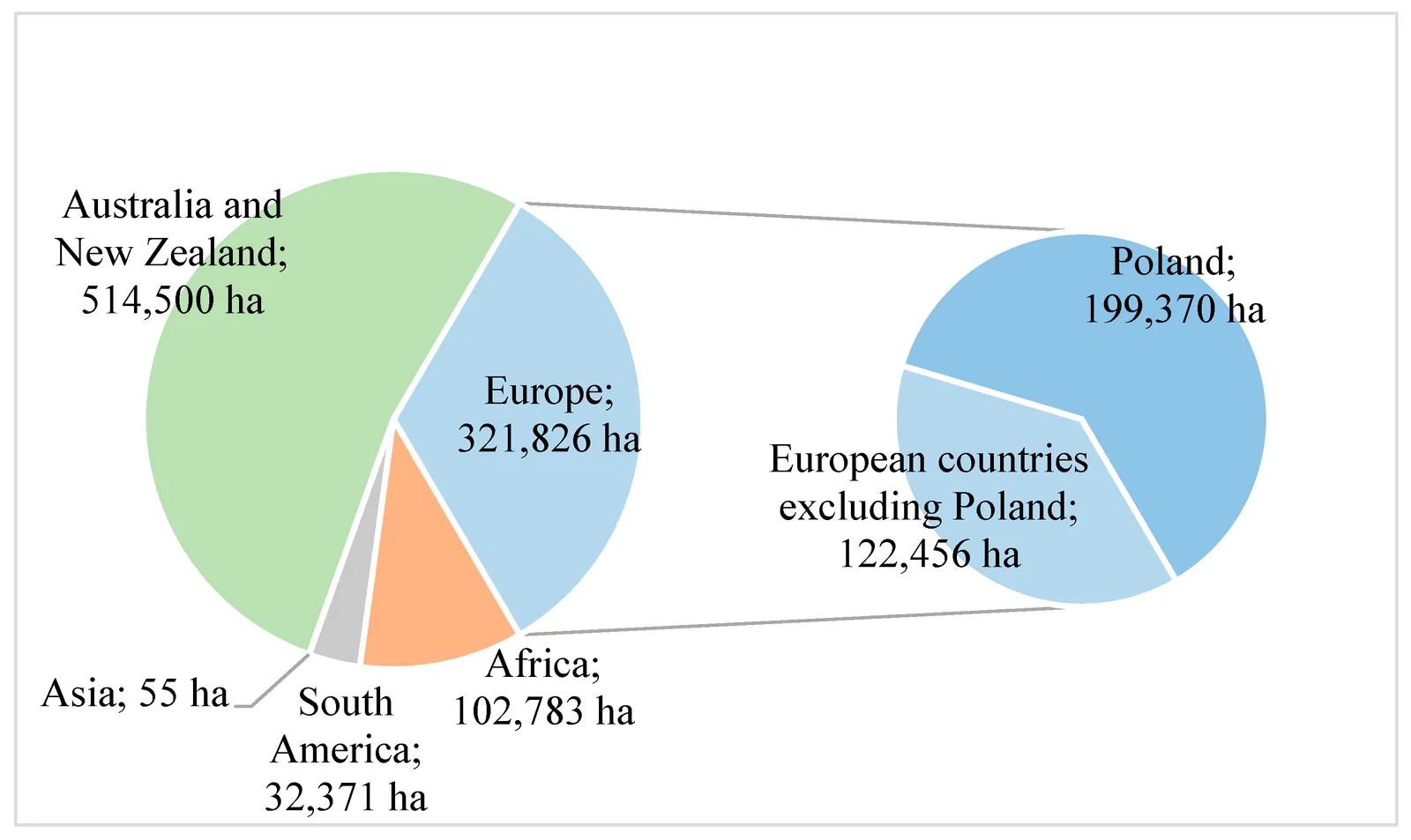
Area of lupin cultivation worldwide in 2024 [15].

In 2024, the area covered by cultivation of lupins amounted globally to 971,536 ha, over 50% of which fell in Australia and New Zealand. In Europe, representatives of lupin species are grown on 321,826 ha, including 199,370 ha in Poland, which accounts for over half of the European crop area. The Food and Agriculture Organization of the United Nations does not mention the lupins cultivation acreage in North America, or *Lupinus* plants are not grown there [15] (Figure 2).

## 2. Scope, Literature Search and Selection

This study is a narrative literature review designed to provide a comprehensive overview of the current knowledge on the chemical composition, biological activities, and applications of *Lupinus angustifolius*. Therefore, systematic review methodology and reporting guidelines (e.g., PRISMA) were not applied.

The aim of this review is to identify the chemical compounds present in narrow-leaved lupin. It also aims to highlight their uses in medicine, cosmetics and the food industry. Based on the information obtained, we are able to describe the potential of narrow-leaved lupin; furthermore, by comparing scientific research with currently available products, we have generated widespread interest in lupins.

A review of the chemical composition was carried out using the Google Scholar search engine. The following combinations of keywords were used: “*Lupinus angustifolius*” or “blue lupin” or “narrow-leaved lupin” and “protein” or “amino acids” or “fatty acids” or “carotenoids” or “phytosterols” or “fiber” or “sugars” or “alkaloids” or “vitamins” or “macroelements” or “microelements” or “flavonoids” or “phenolics” or “volatile compounds”. The section on medical applications was compiled by collating the terms “*Lupinus angustifolius*” or “blue lupin” or “narrow-leaved lupin” and “medicine” or “anticancer”. The clinical trials were found via the website clinicaltrials.gov using “*lupinus*” or “lupin”. Ingredients derived from lupin were found on the website cosmileeurope.eu using “*lupinus*” or “lupin”. Each ingredient was then searched for on the website Google Patents. Google was used to search for cosmetics available on the market, using: “lupin” or “*lupinus*” and “cream” or “skin care” or “lipstick” or “peeling” or “hair care”. To search for products made from lupin or containing lupin, Google Scholar was used with “lupin” or “*lupinus*” and “yoghurt” or “cookies” or “tempeh” or “oil” or “chips” or “coffee” or “milk” or “meat” or “egg” or “bread” or “cheese” or “pasta” or “cakes” or “tofu” or “miso” or “beer” or “flakes” or “snack”. Additional manual searches were carried out by reviewing the bibliographies of relevant review articles and key publications. ChemSketch (Freewere) 2025.2.5. was used to draw the chemical structures. The inspiration for these structures came from the PubChem (pubchem.ncbi.nlm.nig.gov) website and scientific publications in which the chemical structures in question were described.

## 3. Chemical Components

### 3.1. Protein

The narrow-leaved lupin is characterized by a favorable content of protein in the seeds, amounting to 32.8–33.9% of dry mass. For comparison, the protein content in other leguminous plants is as follows: yellow lupin 37.5–40.6%, soya bean 35.4–39.9%, white lupin 34.7–38.9%, pea 23.4–24.7% of dry mass [16]. The highest protein content is typical of the Andean lupin, reaching the level of 41–53% in dry mass [17]. In plants, peptides have different functions: they inhibit insect feeding and participate in defense response, control cell divisions, take part in cell signals, act as protein precursors, control physiological functions and regulate ontogeny [18,19,20,21,22,23,24]. For instance, β-conglutins participate in seed germination, have fungicidal effects, display a protective role against necrotrophic pathogens, and can perform a signaling function in the physiological intracellular processes and in cell adaptation to environmental changes (such as pathogen attacks) [25,26,27]. Four families of storage proteins have been identified in *L. angustifolius* seeds, comprising three unique conglutins α, seven β, two γ and four δ [28], whose percentage share differs (Table 1). The peptide GPETAFLR has been identified in narrow-leaved lupine; it exhibits anti-inflammatory effects [28], reduces osteoclastogenesis [29], has neuroprotective effects [30], and prevents the onset and progression of age-related macular degeneration [31], as well as VPL, PGP and YL PEPTIDES having anti-anxiety and analgesic properties, have been identified [29]. Moreover, pancreatin and pepsin, representing sequences RLGN, NVLSQL, LTFPGSAD and AGGPQQR, which are inhibitors of ACE and DPPIV [30], have been detected. β-conglutins in the narrow-leaved lupin can be perceived as a new source of nutraceutical proteins, which—thanks to their health-promoting properties—are perfect candidates for functional food; however, for them to serve this purpose, their allergic potential should be taken into consideration [31]. Peptides are health-enhancing compounds showing anti-inflammatory, antioxidant, anticancer and antimicrobial properties. Their antioxidant value and wide health-promoting potential alleviate inflammatory diseases such as diabetes type 2, for example [18,31,32,33,34,35].

### 3.2. Amino Acids

**Figure 3:**
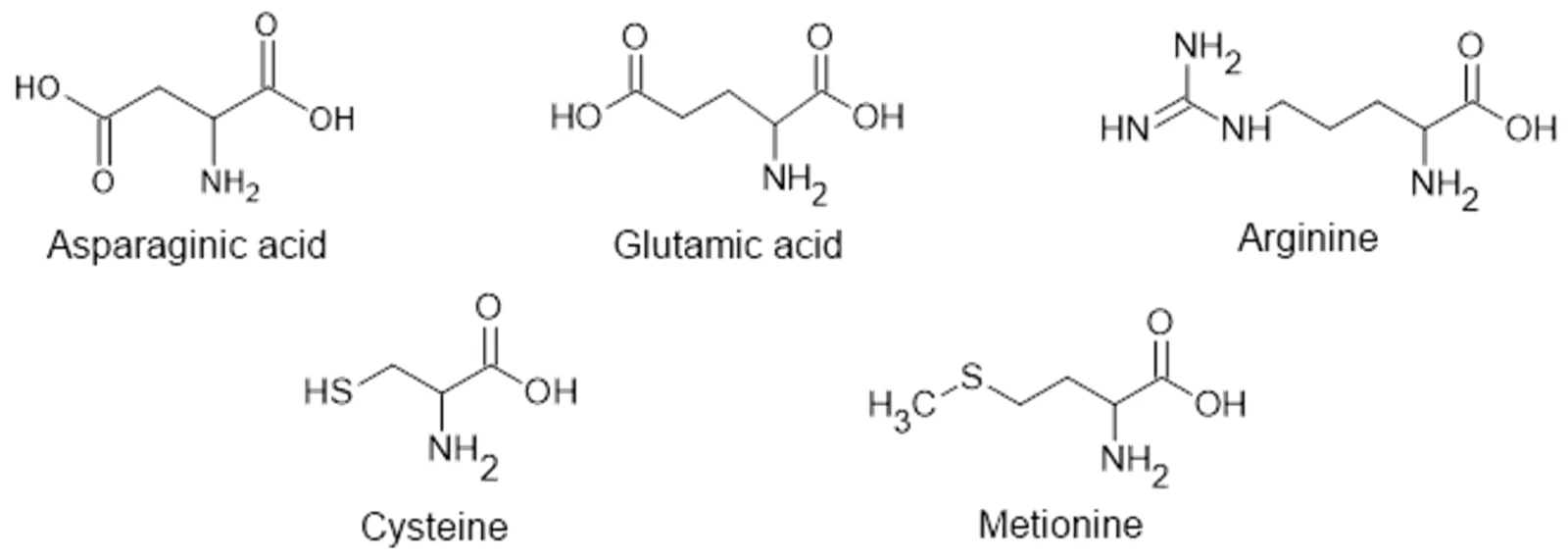
Chemical structures of the amino acids found in *Lupinus angustifolius*.

The amino acid composition of lupin is diverse (Table 1 and Figure 3).

Based on analyses of different species of the *Fabaceae* family, the contents of amino acids in *Pisum sativum*, *Vicia faba* (spring and winter forms), *Lupinus angustifolius*, *Lupinus luteus* and *Glycine max* have been compared. Alanine has been detected at the highest amount in *Pisum sativum* (4.4 g/16 gN) and at a corresponding value in the narrow-leaved lupin (3.4 g/16 gN). Aspartic acid occurs at the highest level in *Pisum sativum* (11.1 g/16 gN), whereas in the narrow-leaved lupin, it figures at 9.5 g/16 gN. Cysteine is recorded at the highest amount in *Lupinus luteus* and *Glycine max*, 1.8 g/16 gN, while in *Lupinus angustifolius*, it reaches 1.5 g/16 gN. The glutamic acid content is the highest in *Lupinus luteus* (21.6 g/16 gN), and in the narrow-leaved lupin, it amounts to 21.1 g/16 gN. The highest level of glycine has been found in *Pisum sativum* (4.4 g/16 gN), while in the narrow-leaved lupin, it is 4.2 g/16 gN. The highest content of proline has been recorded for the soya bean (4.6 g/16 gN), and in *Lupinus angustifolius*, this amino acid reaches the level of 3.7 g/16 gN. Serines occur at the highest level in *Glycine max* (5.1 g/16 gN) and in the white-leaved lupin—4.5 g/16 gN. Tyrosine occurs in the largest amount in the narrow-leaved lupin, 3.8 g/16 gN. Arginine is found at the highest amount in *Lupinus luteus*, 10.4 g/16 gN, whereas in *Lupinus angustifolius*, it reaches 10.1 g/16 gN. Histidine reaches the highest values in *Lupinus angustifolius* and *L. luteus* (2.7 g/16 gN). Isoleucine is recorded at highest amount in *Pisum sativum* (4.4 g/16 gN), whereas in the narrow-leaved lupin, it reaches 4.3 g/16 gN. Leucine reaches at the highest level in *Pisum sativum* (7.2 g/16 gN), while in the narrow-leaved lupin, its content is 7.0 g/16 gN. The lysine content is 7.3 g/16 gN in *Pisum sativum*, whereas it is 4.8 g/16 gN in *Lupinus angustifolius*. The level of methionine is 1.8 g/16 gN in *Glycine max*, while in *Lupinus angustifolius*, it is 0.7 g/16 gN. Phenylalanine occurs at 4.9 g/16 gN in *Pisum sativum* and in *Lupinus angustifolius* at 4.0 g/16 gN. Threonine reaches 4.7 g/16 gN in *Glycine max*, whereas it is 3.5 g/16 gN in the narrow-leaved lupin. Valine is recorded at 4.9 g/16 gN in *Pisum sativum* while in *Lupinus angustifolius* at 4.2 g/16 gN [36].

Amino acids perform an important function in the growth and development of plants. They build proteins, are precursors of metabolites, and are involved in the complex mechanisms of transcriptional and post-transcriptional regulation. They contribute to tolerance to stress, participate in plant responses to different environmental stresses and take part in plant fertilization, the formation of intracellular structures, seed formation and developing immunity [38,39,40,41,42]. The narrow-leaved lupin is characterized by a rich composition of amino acids [37]. Consumption of amino acids by humans stimulates the synthesis of proteins that build the skeletal muscles. Amino acids constitute components for the formation of particles. They are neurotransmitters. They take part in physiological and biochemical processes in the cell. They improve the gut microbiota and the resistance of mucous membranes, which favorably affects the intestines and contributes to the better local and general health of an organism [43,44,45,46].

### 3.3. Fatty Acids

**Figure 4:**
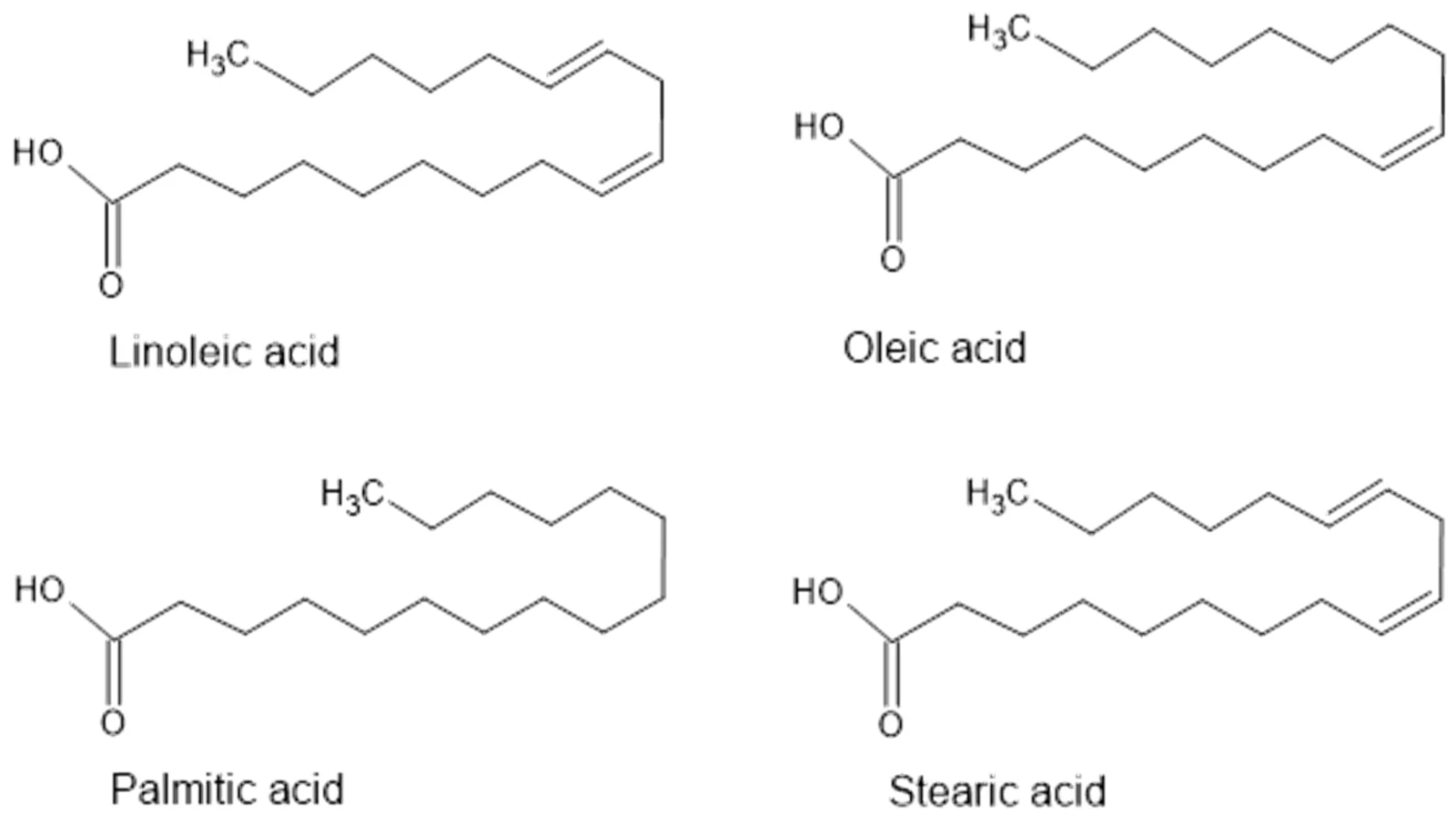
Chemical structures of fatty acids found in *Lupinus angustifolius*.

The profile of fatty acids has been determined for the narrow-leaved lupin, white lupin, yellow lupin, andean lupin, pea, chickpea, lentil, grass pea, common bean and broad bean. Saturated fatty acids (SFAs) have been recorded at the highest level in the narrow-leaved lupin (0.89–1.11 g/100 g) and andean lupin (2.81 g/100 g). *Lupinus angustifolius* contains 9.76–13.03% medium-chain (C14–C16) fatty acids, which points to its dominance over other studied plants in this respect. In the narrow-leaved lupin, long-chain (≥C18) fatty acids occur within the range of 83.82–89.16 g/100 g, whereas in the white lupin, for instance, they reach 91.18–92.17 g/100 g. The narrow-leaved lupin possesses a high content of oleic acid (OFA), i.e., 9.64–12.84 g/100 g, in comparison with other species representing the genus *Lupinus*, except for the andean lupin, where the oleic acid reaches a similar level of 12.41 g/100 g. The atherogenicity index of all the investigated fabaceans is low [47]. The contents of particular fatty acids in the narrow-leaved lupin seeds vary depending on the genotype and ecotype (Table 2 and Figure 4) [48,49].

Pertinent studies indicate that—compared with the white and yellow lupins—the content of saturated fatty acids—C13 (tridecanoic acid), C14 (myristic acid), C15 (pentadecanoic acid), C16 (palmitic acid), C17 (margaric acid), C18 (stearic acid), C23 (tricosanoic)—is statistically higher in the narrow-leaved lupin, whereas that of unsaturated fatty acids is statistically lower than in the other lupin species mentioned [53].

Fatty acids represent components for lipid membranes, the composition of which ensures that various functions can be performed. They regulate the properties of biological membranes, which is reflected in their being more or less fluid. In general, changes in membrane lipids contribute to the plants’ ability to acclimatize and play an important role in the course of the photosynthetic process. Lipids take part in the response of plants to biotic and abiotic stress, and can participate in interspecific communication and defense reactions in plants [54,55,56,57,58,59,60,61,62].

In humans, saturated fatty acids can be responsible for inflammatory conditions and can contribute to mortality [48,63]. Replacement of saturated acids with unsaturated ones reduces the risk of developing coronary heart disease, whereas replacement with linoleic acid also decreases the risk of death due to coronary heart disease. Leguminous plants, rich in fatty acids, have a functional quality for human health [64,65]. The fatty acids coming from the seeds of fabaceans point to the high protective potential of these plants in the prevention and treatment of diet-related disorders. Taking into consideration these properties, the seeds of leguminous plants can again be recovered as a valuable component of a healthy human diet [47].

### 3.4. Carotenoids

**Figure 5:**
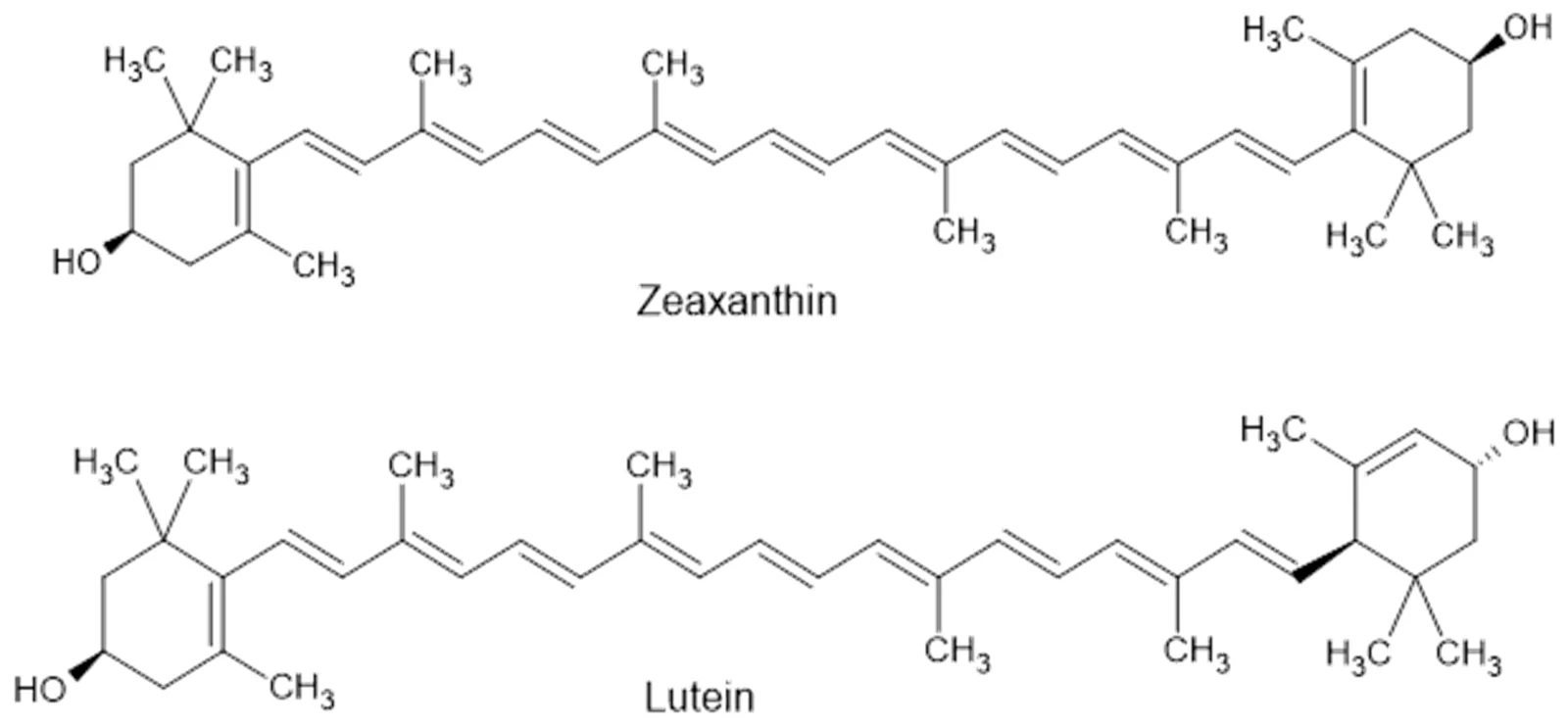
Chemical structures of carotenoids found in *Lupinus angustifolius*.

Carotenoids have been identified in the seeds of *Lupinus angustifolius* (Table 3 and Figure 5).

These are predominantly: violaxantin, lutein, zeaxantin and β-carotene [52,66,67]. In comparison with other lupins, the total content of carotenoids is the highest in the narrow-leaved lupin, ranging from 17.20 to 62.52 mg/kg, depending on the cultivar. In the white lupin, the content of carotenoids ranges between 8.94 and 12.70 mg/kg, whereas in the yellow lupin, it amounts to 6.12–6.33 mg/kg [67]. Depending on the cultivar, the lutein content in the narrow-leaved lupin reaches 35.95 mg/kg. In the white and yellow lupin, it is decidedly lower: 6.65–8.77 mg/kg and 4.30–4.50 mg/kg, respectively [67]. The literature data pertaining to the content of lutein in the soya bean indicate that the amount of this carotenoid in the seeds can vary between 1.35 and 32.08 mg/kg [68,69]. These are lower figures than the values reported for the most lutein-productive cultivars of *Lupinus angustifolius*.

Carotenoids are synthesized by all photosynthetic organisms. The synthesis and storage of carotenoids take place in plastids [70]. Carotenoids and apocarotenoids play important functions in the physiological processes occurring in plants, such as: construction of photosystems, collection of light for photosynthesis, photoprotection, regulation of growth and development. They perform key functions in the detection and signalization of oxidative stress, photosynthesis, photomorphogenesis, photoprotection, and protection against light and UV radiation; they are antioxidants. Carotenoids act as auxiliary pigments, accumulating light in the photosynthetic process. They are involved in the mechanism of resistance to stress and display antioxidative properties [71,72,73,74,75,76,77,78,79,80,81,82]. Pigments, among which there are carotenoids, are responsible for the color of flowers and fruit, which helps attract pollinators and seed spreaders [83,84,85,86,87,88]. Carotenoids present in food are essential to the condition of the eyes, brain and cardio-vascular system. They exhibit antioxidative, antiviral, antileukemic, anticancer, anti-inflammatory and neuroprotective effects. Moreover, they reduce the risk of developing diabetes type 2 [89,90,91,92,93,94,95,96,97,98,99].

### 3.5. Phytosterols

**Figure 6:**
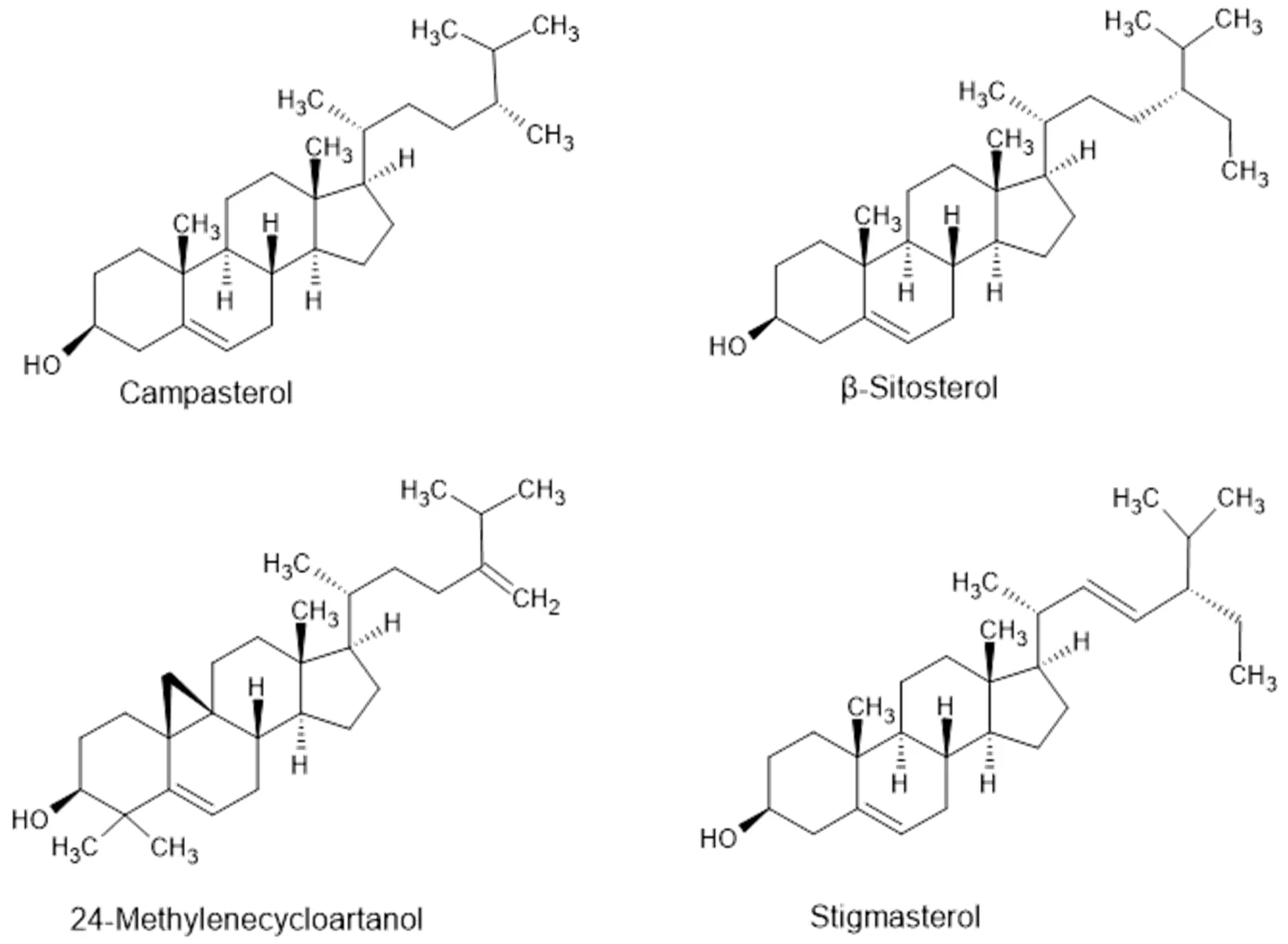
Chemical structures of phytosterols found in *Lupinus angustifolius*.

The total content of phytosterols (Table 4 and Figure 6) in the narrow-leaved lupin seeds ranges from 13,603.6 to 16,007.4 mg/kg. For the sake of comparison, in the white lupin, these values are at the level of 7117.2–11,491.2 mg/kg, and in the yellow lupin, at 13,434.0–15,655.2 mg/kg [52].

In plants, phytosterols are components of cellular membranes and lipid drafts, participate in homeostasis, represent a storage pool of sterols, and perform a key role in different physiological and biochemical processes during the plant development and building up of the resistance of plants to stress [101,102]. Phytosterols diminish the absorption of cholesterol in the intestines, show anti-inflammatory and antioxidative properties, and also are crucial in the antidiabetic diet [103,104].

### 3.6. Fiber and Sugars

The polysaccharides, which build the wall of a cell, constitute dietary fiber. Among the major compounds that compose the cellular wall are: pectins, hemicellulose, cellulose and lignins [105,106]. The total content of dietary fiber in different leguminous plants is as follows: soya bean 108.0–114.9 g/kg, pea 54.4–63.3, white lupin 112.6–120.6, narrow-leaved lupin 159.3–149.0, yellow lupin 128.3–131.4 g/kg [16]. And thus the narrow-leaved lupin is characterized by the highest content of fiber. In this lupin, a fibrous fraction, which is composed of non-starch polysaccharides, neutrally detergent fiber, acid detergent fiber, raw fiber, hemicellulose and cellulose, has been identified [107]. Furthermore, oligosaccharides (sucrose, galactopinitol, galactoinositol, raffinose, digalactopinitol, digalactoinositol, stachyose, verbascose) and non-starch polysaccharides (rhamnose, fucose, arabinose, xylose, mannose, galactose, glucose, uronic acids) have also been recognized (Table 5) [107].

Fiber is present in all plant cells. Its most significant function manifests itself in the mechanical properties of plants, resulting in the cellular wall being thin, strong and flexible. The wall gives a cell its proper shape, constitutes a structure participating in the transfer of information within an organism and with the environment, and also represents a protective barrier against external threats. Forming the interface between adjacent cells, plant cell walls often contribute importantly to the intercellular communication. Due to its surface location, the plant cell wall plays an important role in plant–microbe interactions, including defense responses against potential pathogens [110,111,112,113]. The presence of fiber in the feed of monogastric animals has so far been considered as an antinutritional factor. However, the trend to incorporate it into the diet of non-ruminants has recently been growing in view of the advantages of fiber to the intestinal health of these animals [114], except for poultry [115]. Investigations indicate that fiber affects positively the digestive system of pigs, dogs and cats [116,117]. Moreover, consumption of dietary fiber is conducive to human health through the reduction of the risk of diseases, including metabolic disorders [118,119,120,121].

#### Oligosaccharides from the Raffinose Group

**Figure 7:**
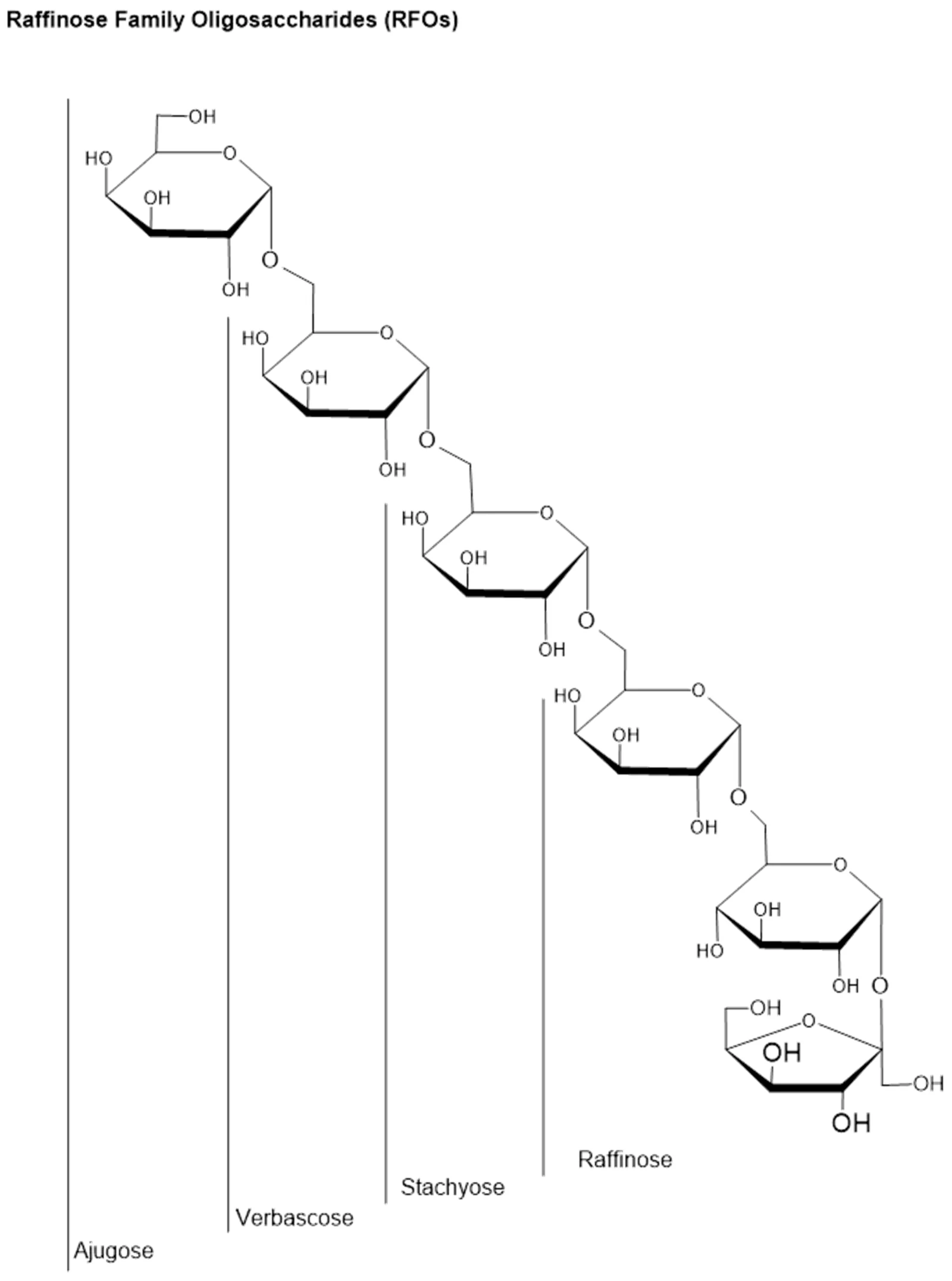
Chemical structures of raffinose family oligosaccharides found in *Lupinus angustifolius*.

Depending on the cultivar, α-galactosides such as raffinose, stacchiose and verbascose have been distinguished at various concentrations in lupin seeds (Table 5 and Figure 7) [108].

The content of oligosaccharides from the group of raffinoses has been found to be different in particular leguminous plants. The highest content of verbascose is characteristic of the broad bean—38.5 mg/g, the greatest level of stachyose is recorded for the white lupin—84.5 mg/g, whereas the yellow lupin has the highest raffinose content—11.5 mg/g. The narrow-leaved lupin is characterized by the lowest content of oligosaccharides representing the raffinose group in comparison with the white and yellow lupins [122]. Ajugose, an oligosaccharide from the raffinose group recognized in leguminous plants, is often taken out of account. Research demonstrates that it is absent from the seeds of the pea, soya bean, common bean, cowpea, lentil, chickpea, green gram, black gram, rapeseed, barley, wheat, Pisum sativum, Brassica campestris, Brassica napus, Brassica nigra and Hordeum vulgare. However, compared with other crop species, ajugose occurs at high levels in lupins. Its largest contents have been detected in the yellow and narrow-leaved lupins, with the lowest values being recorded for the white and andean [109,123,124].

The oligosaccharides representing the raffinose group can have a wide range of functions in plants, as they play a key role in plant physiology and cellular functions such as signal transfer, for instance [125]. They can be a form storing energy and transporting carbohydrates. They take part in fighting both biotic and abiotic stress. They stabilize the cell membrane, show antioxidant properties and participate in the germination of seeds [126,127,128,129,130,131]. Since they are not digested but used by intestinal microbiota, the raffinose group oligosaccharides are responsible for flatulence [132]. Nevertheless, they exert a favorable influence on the gut flora. And therefore, they are recommended for consumption in order to prevent digestive system cancers. Also, they have numerous other biological functions, both in humans and animals, such as antiallergic, anti-obesity, prebiotic and antidiabetic effects or the prevention of non-alcoholic fatty liver disease. Moreover, they display cryoprotective properties [128,131,133].

### 3.7. Alkaloids

**Figure 8:**
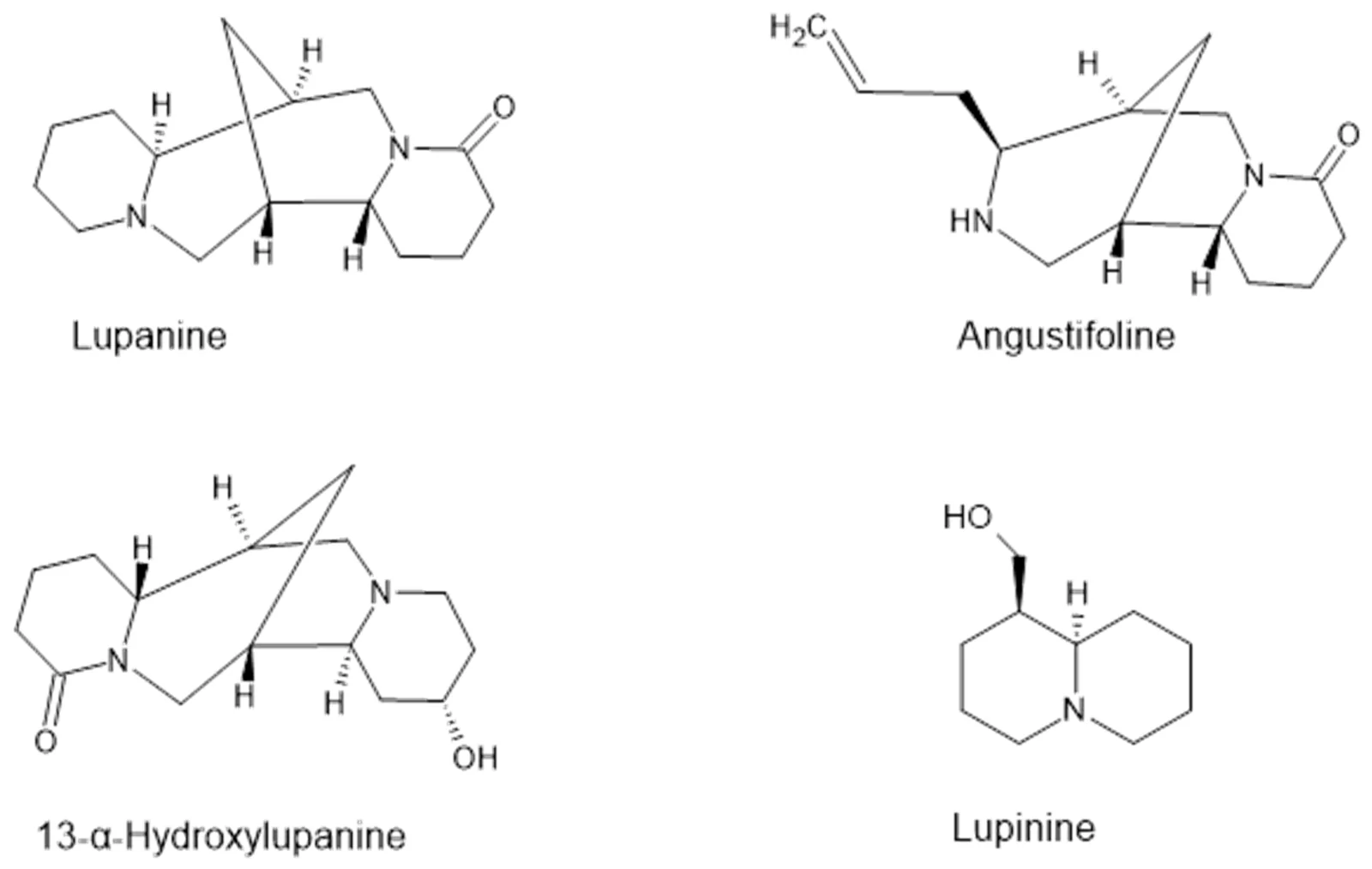
Chemical structures of alkaloids found in *Lupinus angustifolius*.

Alkaloids have been identified in the narrow-leaved lupin above-ground parts and seeds (Table 6 and Figure 8), their content ranging from 8.0 mg/kg up to even 100,000.0 mg/kg, depending on the genotype [134,135,136].

Other data report a level of 0.0005–2.8752% dry seed weight [138]. Predominance of 13α-hydroxylupanine is characteristic of *Lupinus angustifolius*, both its sweet and bitter cultivars. Moreover, angustifoline, lupinine and lupanine occur in increased amounts in the seeds of this species [134,137]. Other studies indicate that 13-OH-lupanine, 11,12-seco-12,13 didehydromultiflorine and lupinine are not present in narrow-leaved lupin [139]. Currently, the narrow-leaved lupin breeding is focused mainly on the creation of cultivars producing low-alkaloid seeds so that this particular species can more effectively be used. A good approach would be to acquire “bitter/sweet” cultivars combining high alkaloid content in vegetative organs while a low level of alkaloids in the seeds, to be attained through control of the synthesis/transport of alkaloids within the plant. The outcome would be acquisition of seeds, which could be used for the purpose of human and animal feeding without the plants losing their adaptive potential due to the elimination of alkaloids, particularly in respect to immunity to pathogens [6]. In plant organisms, alkaloids play a significant protective role against herbivores—insects, vertebrates, and arthropods—as well as against pathogens, and they also act as growth regulators. They store nitrogen and also take part in the protective mechanism triggered by abiotic stressors (heavy metals, salination, temperature, drought, reactive forms of oxygen, free radicals, nutritional deficiency, etc.). Alkaloids belong to compounds that participate in allelopathy, among others, by hindering germination of other species’ seeds. Furthermore, they can be present in nectar to repel undesirable fertilizers, and exhibit an antiviral effect [140,141,142,143,144,145,146,147,148].

Although—in general—alkaloids are disadvantageous when in human food and animal feed, their biological activity can be of important use in pharmacy. The quinolizidine alkaloids, for example, display anthelmintic action, a cytotoxic effect, antiviral and antimicrobial action, an insecticidal effect, and antimalarial and anti-acetylcholinesterase action [149,150,151,152,153,154,155].

### 3.8. Vitamins

**Figure 9:**
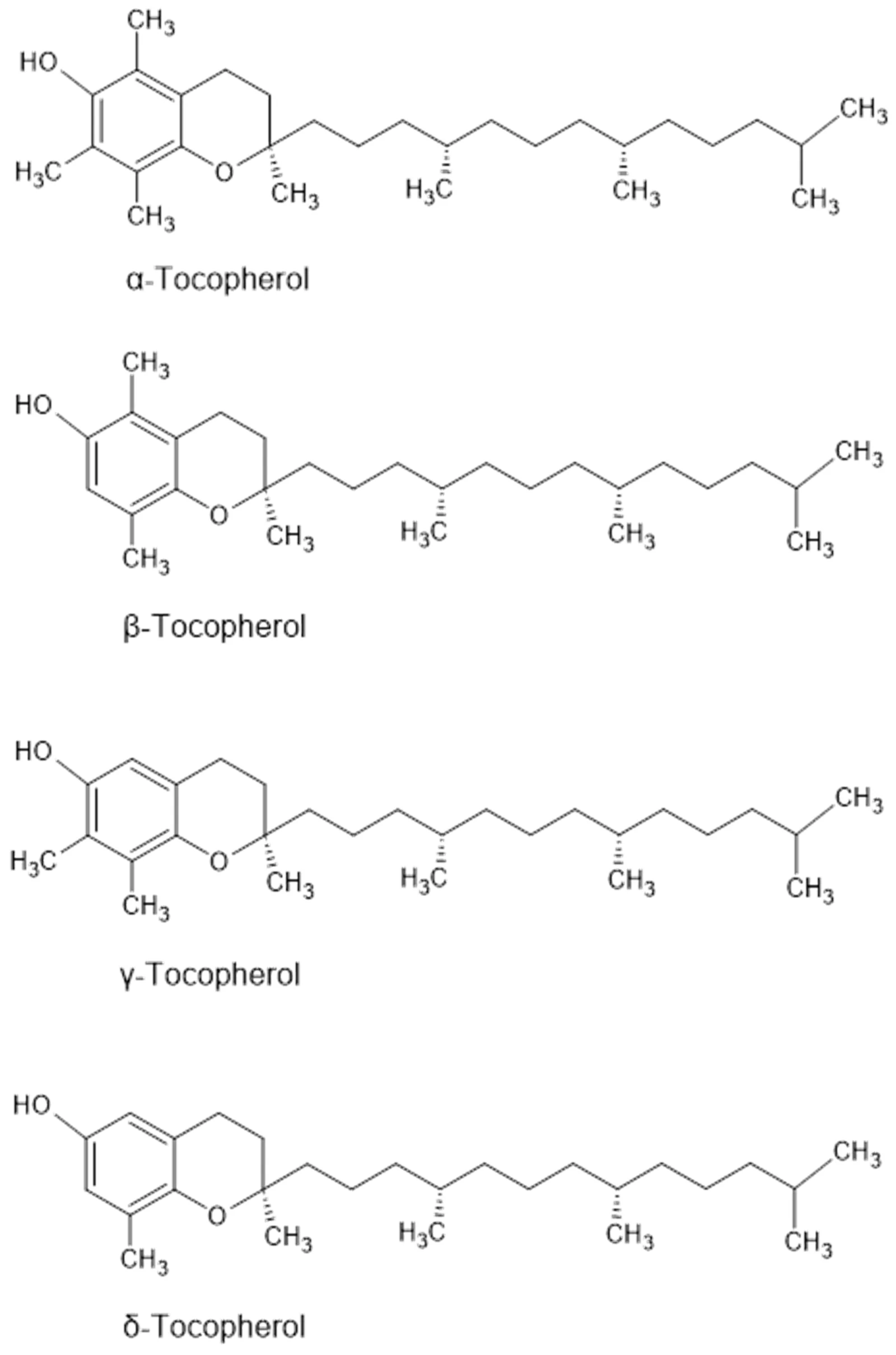
Chemical structures of vitamin E found in *Lupinus angustifolius*.

In the narrow-leaved lupin, vitamins from group B and E have been identified (Table 7 and Figure 9) [108].

Thiamine (vitamin B1) is present at a level of 5.66–7.12 mg/kg, while riboflavin (vitamin B2) is at 2.36–2.74 mg/kg. These figures correspond with those recorded for the soya bean (thiamine: 9.12 mg/kg, riboflavin: 3.2 mg/kg) and bean (*Phaseolus vulgaris*) (thiamine: 4.6–5.0 mg/kg, riboflavin: 1.5–1.6 mg/kg) [157,158]. Vitamin E occurs in *L. angustifolius* in various amounts: α-tocopherol (1.42–8.61 mg/kg), β-tocopherol (2.23–3.12 mg/kg), γ-tocopherol (10.30–91.52 mg/kg), and δ-tocopherol (1.26–1.41 mg/kg) (Table 7), whereas in other fabacean plants, these values are as follows: *Pisum sativum*: α-tocopherol 0.7–1.46 mg/kg, γ-tocopherol 56.5–133.6 mg/kg, δ-tocopherol 1.6–8.9 mg/kg; *Phaseolus vulgaris*: γ-tocopherol 27.3–48.5 mg/kg, δ-tocopherol 0.74–1.55 mg/kg; *Vicia faba*: α-tocopherol 4.87–8.36 mg/kg, γ-tocopherol 47.4–56.0 mg/kg, δ-tocopherol 0.9–1.0 mg/kg; *Cicer arietinum*: α-tocopherol 17.6–21.1 mg/kg, γ-tocopherol 64.4–86.0 mg/kg, δ-tocopherol 4.7–5.9 mg/kg; *Lens culinaria*: α-tocopherol 2.5–4.0 mg/kg, γ-tocopherol 37.2–50.0 mg/kg, δ-tocopherol 0.5–0.6 mg/kg; *Glycine max*: α-tocopherol 7.80–8.0 mg/kg, γ-tocopherol 81.7–89.8 mg/kg, δ-tocopherol 41.9–43.8 mg/kg; *Lupinus albus*: α-tocopherol 0.67–1.65 mg/kg, γ-tocopherol 61.2–131.3 mg/kg, δ-tocopherol 1.1–2.5 mg/kg [156].

In plants, vitamins perform various biological functions. Among others, they constitute indispensable components of some enzymes for their proper acting; they are used in the whole cell as antioxidants, co-factors and substrates of cellular processes; as well as they play an essential role in the response reaction to different types of environmental stress [159,160,161,162,163]. Human organisms require these compounds to be present for correct functioning. Certain indispensable nutrients such as vitamins cannot be produced by a human organism and therefore ought to be acquired from food [164,165].

### 3.9. Macroelements and Microelements

Macro- and microelements are indispensable chemical elements, without which a plant would not be able to grow and develop in the proper way [166]. In the seeds of the narrow-leaved lupin, the following macroelements have been identified: Na, Mg, K and Ca, and microelements as follows: Mn, Fe, Zn, Cu, Ni, Cr, Se, Co, Sr, Al, Pb, Cd, As, Ag (Table 8) [49].

For leguminous plants, such as the soya bean; pea; faba bean (*Vicia faba*); narrow-leaved, white and yellow lupins; among others, contents of the main macroelements have been determined. The lowest content of phosphorus has been found in the white lupin seeds (3.50–3.88 g/kg) and the highest in the yellow lupin seeds (6.90–7.10 g/kg). Potassium occurred at the lowest level in the pea (7.4–9.2 g/kg), whereas its highest content has been noted for the yellow lupin seeds (11.2–11.5 g/kg). The faba bean (*Vicia faba*) seeds are characterized by the lowest content of magnesium (0.99–1.16 g/kg), while the highest amounts of this element have been detected in the soya bean (2.12–2.25 g/kg) and yellow lupin seeds (2.15–2.22 g/kg). A low content of calcium is characteristic of the pea seeds (0.28–0.35 g/kg), whereas in the narrow-leaved lupin, the content of this element has been found at the highest level (1.59–2.08 g/kg) in comparison with the other above-mentioned leguminous plant species [16].

Microelements perform various functions in plants. They fall within the composition of functional enzymes and take part in photosynthesis, metabolism, and the transport of nutrients in the cell. They are engaged in the symbiotic relation with *Rhizobium* bacteria and participate in the synthesis of the cell wall and other structural elements [167,168,169,170]. In people, microelements are of importance in metabolic paths. They participate in the development of certain tissues, perform a transporting role, take part in maintenance of the hormonal and enzymatic balance, and contribute to the proper functioning of the immune system [164,166,167,171,172,173]. Research pertaining to contents of the principal microelements in the seeds of the soya bean; pea; faba bean; and narrow-leaved, white and yellow lupins reveals differences. The content of iron is the lowest in the narrow-leaved lupin seeds (30.1–31.1 mg/kg) whereas the highest in those of the soya bean (61.0–99.2 mg/kg). Manganese has been recorded at the lowest level in the pea (6.1–9.2 mg/kg), while the highest values are recorded for the white lupin seeds (474.8–636.9 mg/kg). The lowest content of zinc has been determined in the narrow-leaved lupin (20.8–22.0 mg/kg), and the highest values recorded for the yellow lupin (46.2–48.1 mg/kg). Copper occurred at the lowest level in the narrow-leaved lupin (3.5–3.8 mg/kg), while the highest content of this microelement has been found in the soya bean seeds (11.1–14.3 mg/kg [16].

Macroelements are important factors in the growth and development of plants. They constitute structural units and take part in the processes of signaling; in the creation, stabilization and defining of the properties of membranes; and in the synthesis of proteins and the activity of enzymes. They affect the operation of stomata and the photosynthetic process, and regulate numerous responses of cells to different stimuli [167,169,174,175,176]. Macroelements are made use of by a human organism during the whole life. They maintain physiological functions and the acid–base balance, and are indispensable for the synthesis of organs and their regulation, as well as the functioning of cells. They are engaged in the stabilization of cellular membranes, the hormonal system, and the transport of particles. Also, they control enzymes and proteins, act as co-factors, and participate in the transmission of stimuli in nerves [165,167,174,177,178,179,180].

### 3.10. Flavonoids

**Figure 10:**
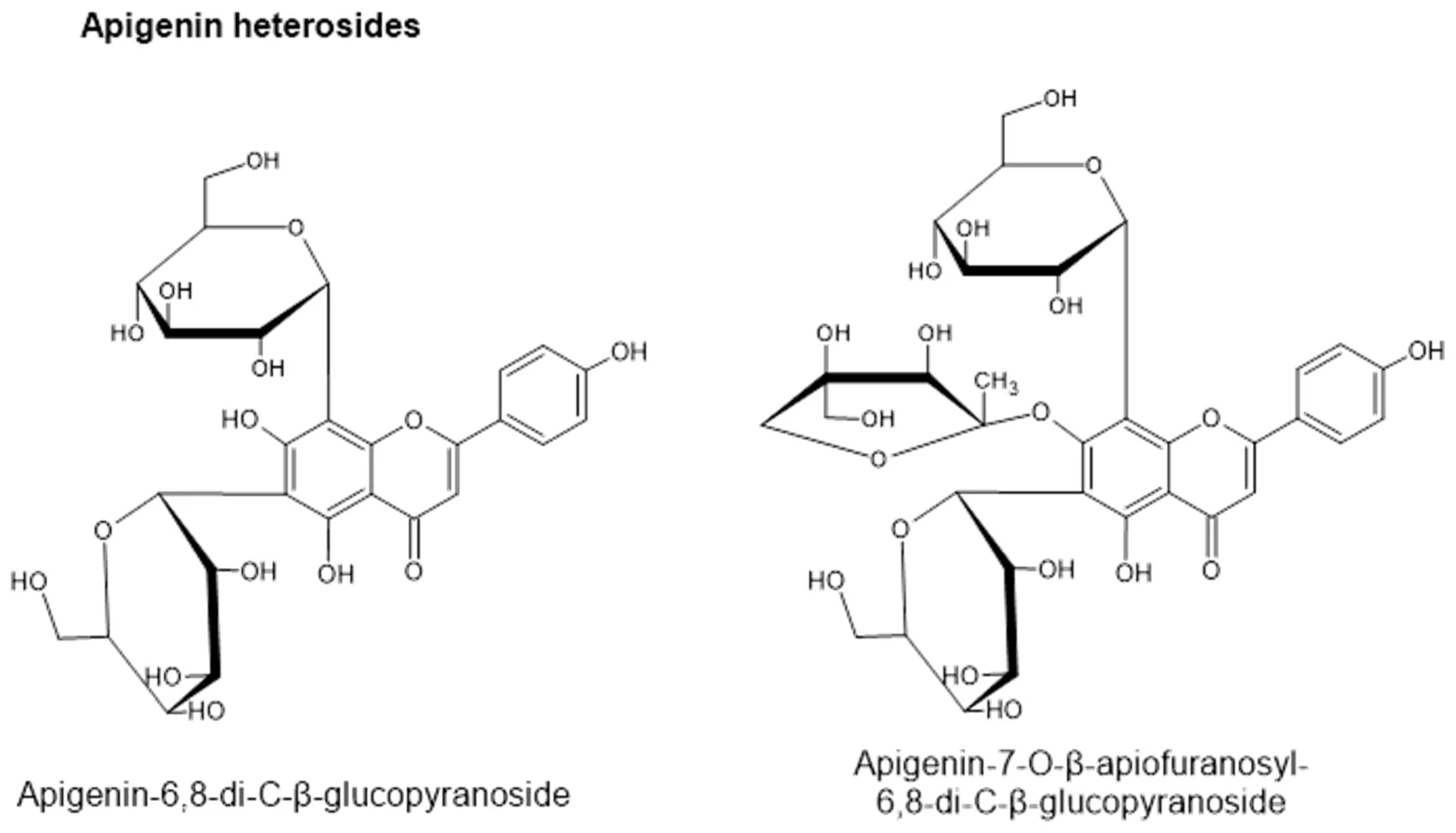
Chemical structures of apigenin heteroside found in *Lupinus angustifolius*.

Numerous flavonoids and their derivatives, as well as glycosides of these compounds, have been identified in the narrow-leaved lupin (Table 9 and Figure 10) [181,182].

The greatest values are recorded for apigenin heteroside 665.9–692.0 mg/kg [184], apiin 0.12–0.19 mg/kg, luteolin 1.23–2.31 mg/kg, hesperidin 0.19–0.29 mg/kg, apigenin-7-glc 0.17–0.25 mg/kg, and naringenin 0.03–0.05 mg/kg. The total content of flavonoids in the *Cicer arietinum* amounts to 27.5–127.8 mg/kg, in *Pisum sativum* seeds to 96.6–226.7 mg/kg, and in *Faba bean* seeds to 28.8–366.7 mg/kg. In lupins, the only flavonoid detected is apigenin heteroside, whose content reaches 130.0–465.2 mg/kg in the white lupin, 665.9–692.0 mg/kg in the narrow-leaved lupin and 706.0–1143.7 mg/kg in the yellow lupin seeds [184]. In the soya bean, the flavonoid content reaches the level of 0.68–2.13 mg/kg [187].

In plants, flavonoids participate in the scavenging of reactive oxygen species (ROS) and free radicals, and in the antioxidant mechanism. They are involved in the response to environmental and photooxidative stress, regulate plant development and interactions with commensal microbes, act as biostimulators, induce immunity to biotic and abiotic stress, contribute to the communication with rhizosphere microbes, participate in allelopathy and constitute signaling compounds that could protect the plant against herbivores [188,189,190,191,192,193,194,195,196,197,198]. For a human organism, flavonoids display anti-inflammatory, antimicrobial, antioxidant and anticancer properties. They protect the cardio-vascular system; have antibacterial, antifungal, antiviral, antimutageneous, neuroprotective and cardioprotective activity; and also act against antibiotic-resistant bacteria [199,200,201,202,203,204].

### 3.11. Other Phenolic Compounds

**Figure 11:**
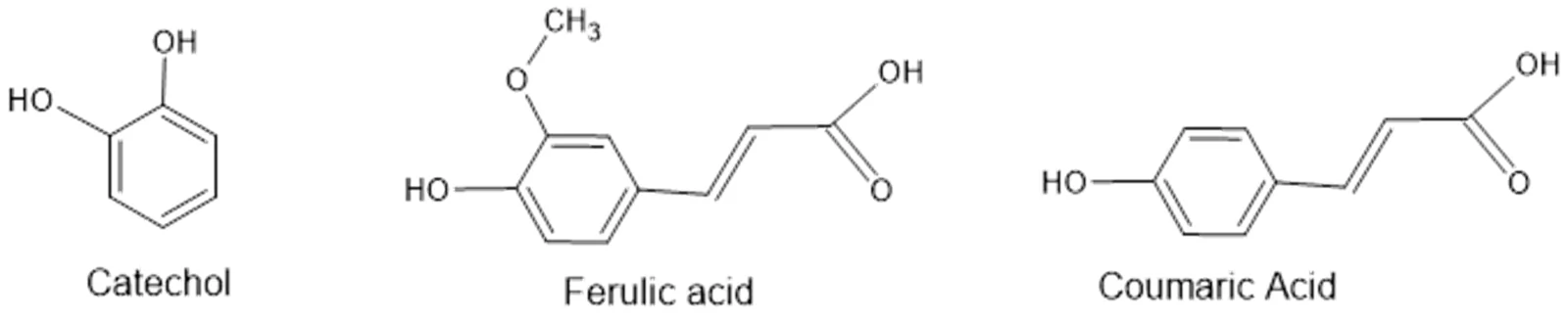
Chemical structures of phenolic compounds found in *Lupinus angustifolius*.

In the seeds of different ecotypes of fabacean species, an array of phenolic compounds have been identified. They represent the following groups: hydroxycinnamics, phenylpropanoids, benzoic acids, hydroxybenzoic (Table 10 and Figure 11) [50,182,186].

In *Cicer arietinum*, the content of phenolic acids in the seeds is within the range of 27.5–107.0 mg/kg, in *Pisum sativum* 57.5–248.2 mg/kg, and in *Vicia faba* 22.9–138.2 mg/kg. In the white lupin, phenolic acids have been detected only in one cultivar at 7.5 mg/kg. They occur at the level of 10.0–27.6 mg/kg in the yellow lupin, whereas in the narrow-leaved lupin, no phenolic acids have been recognized [184].

Phenolic compounds play an important role in the growth and development of plants and in their protection mechanism. They possess antioxidant properties, neutralizing the consequences of oxidative stress. Phenolic compounds may repel herbivores and can constitute substrates in the synthesis of other compounds. They protect the plant organism against UV radiation and have protective character in general. They participate in the creation of mycorrhizal symbiosis and the formation of bacterial warts. Additionally, they generate the mechanism of protection against harmful microbiota and chelating transport factors. Moreover, they can be sex hormones and cell differentiation effectors. Apart from that, the release of phenolic compounds mostly accounts for the defense mechanism against foraging herbivores and allelopathic interaction [205,206,207,208,209,210].

In humans, phenolic compounds prevent inflammatory processes, neurodegenerative diseases, cancers and cardio-vascular disorders. They display antimicrobial, antioxidant, anti-parasite and anticancer potential. As antioxidants, these compounds can potentially be effective in the treatment of various disorders concerning the epidermis, including aging symptoms, and act as protective agents against neurodegenerative diseases. They can be used for regulation of the immunological response of the intestinal mucosa and in the treatment of allergies [207,211,212,213,214,215,216,217,218,219].

### 3.12. Volatile Organic Compounds and Terpenoids

**Figure 12:**
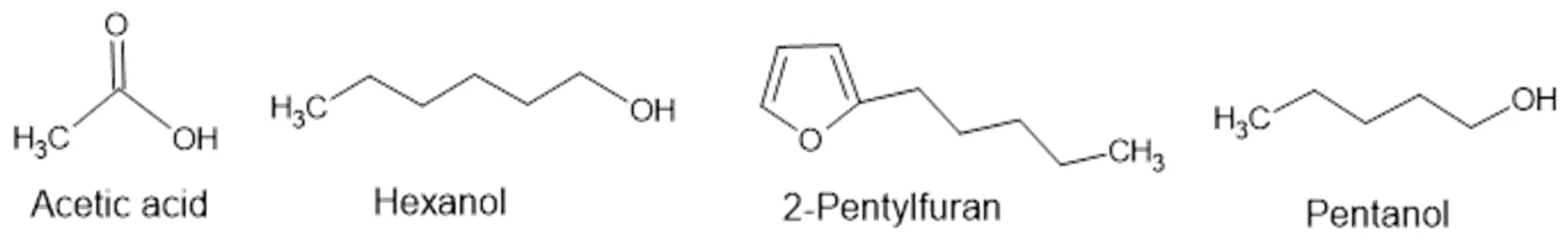
Chemical structures of volatile compounds found in *Lupinus angustifolius*.

A wide range of volatile compounds representing different terpene groups and their glycosides have been identified in the seeds of *Lupinus angustifolius* (Table 11 and Figure 12) [50,66,220,221,222,223].

One of the more interesting volatile compounds detected in the narrow-leaved lupin is lupeol (29.58–87.66 mg kg^−1^) [50]. In plants representing other families, its content has been recorded at higher concentrations, e.g., *Mangifera indica* 0.124 g/100 g [224], *Derris scandens* 32.79 mg/100 g, *Albizia procera* 21.44 mg/100 g, *Diospyros rhodocalyx* 40.72 mg/100 g [225]. Lupeol exhibits anticancer, anti-inflammatory, and antioxidant properties; protects the cardio-vascular system; acts as an anti-microbiota agent, also protectively on the skin; and favorably affects chronic illnesses [226,227,228].

Volatile compounds occurring in plants play an important role in the communication with other plants and with pollinators, pests and herbivores. They contribute to allelopathy, attract seed-spreaders, and protect the plant’s underground and above-ground parts against herbivores and pathogens. Apart from that, they protect plants against abiotic stress, namely, intense light, temperature or oxidative stress [229,230,231,232,233,234,235,236,237,238,239,240]. Aroma—scent—belongs to traits that are essential for consumers when shopping, and therefore, it is an important factor encouraging the purchase of an article. Volatile compounds have potential in the regulation of appetite [241,242]. The negative approach to legume food results from the undesirable smell of fabacean seeds. Detailed identification of the odor-active compounds in leguminous plants could enable determination of their direct origin in order to elaborate strategies aimed at diminishing their recognition [243]. It is worth mentioning that volatile chemical compounds show numerous biological properties: antiviral, antimicrobial, antifungal or antibacterial [244,245,246,247,248,249].

### 3.13. Summary of the Compounds Found in Narrow-Leaved Lupin

As summarized in Table 12, narrow-leaved lupin exhibits a distinctive nutritional profile that differentiates it from other economically important grain legumes. Although its protein content (32.8–33.9% DM) is slightly lower than that of soybean and other lupin species, it remains substantially higher than that of pea, confirming its value as an important plant protein source. More importantly, narrow-leaved lupin is characterized by the highest dietary fiber content among the compared legumes, exceeding soybean, white lupin, yellow lupin, and especially pea. This exceptionally high fiber content enhances its nutritional value while also supporting gut health and metabolic benefits.

Beyond its macronutrient composition, narrow-leaved lupin is particularly rich in several health-promoting phytochemicals. It contains the highest total carotenoid concentration among the compared lupin species and accumulates markedly higher levels of lutein than white and yellow lupins, with concentrations that may even exceed those reported for soybean cultivars. In addition, narrow-leaved lupin is one of the richest sources of phytosterols and contains remarkably high levels of apigenin heterosides, compounds recognized for their antioxidant and anti-inflammatory activities. The species also exhibits the highest calcium concentration among the legumes compared, although its iron, zinc, and copper contents are relatively lower than those reported for soybean and some other legumes. Overall, the combination of high-quality protein, exceptionally high dietary fiber, elevated carotenoid and phytosterol contents, and abundant bioactive flavonoids makes narrow-leaved lupin a particularly attractive crop for functional foods, nutraceutical applications, and the development of health-promoting plant-based products.

## 4. Usefulness of Lupins

### 4.1. Potential Anticancer Quality of Lupins

Extract from lupin seeds, containing rosmarinic acid, quercetin and ergosterol, displayed the highest anticancer potential among the tested preparations against the colorectal carcinoma Caco-2 cell line [100]. Extracts from the seed coat of *L. angustifolius* and *L. albus* induced apoptosis in the cells of MIA PaCa-2 (human pancreatic cancer). They contained quinolizidine alkaloids at concentrations below the toxicity level for people, but with potential benefits for human health [250]. Investigations on extracts from *L. angustifolius* roots and shoots reveal they could be taken into account as measures in the primary and secondary prevention of cancer. They display an anticancer receptor-positive and receptor negative effect on cell lines of breast cancer [251]. Extracts from the narrow-leaved and white lupin seeds inhibit cellular cycles through inducing apoptosis of colorectal carcinoma cells via increased production of ROS and activation of caspases-3/7 [252]. Consumption of fiber from the narrow-leaved seeds was found to improve some markers of proper bowel function and markers of colorectal cancer risk [253]. Other latest studies deny these reports, though. A set of in vitro tests were applied to examine the properties of alkaloids occurring in lupins: angustifoline, lupanine, 13-hydroxylupanine, lupinine and sparteine. The outcome was that none of the compounds showed cytotoxic, genotoxic or mutagene potential when in vitro [254]. The proteins of β-conglutins from the narrow-leaved lupin seeds show a cytotoxic effect on breast cancer cells when at very low concentrations [255].

### 4.2. Clinical Studies of Lupin Benefits on Health

In 2014, a hypothesis was put forward about the lupin fiber activity reducing the lipid level in blood, which can mainly be attributed to the formation of short-chain fatty acids (SCFAs) [256]. Clinical studies indicate that incorporation of lupin protein into a diet decreases the levels of total cholesterol, LDL, triacylglycerols, homocysteine and uric acid in patients with hypercholesterolemia [257]. Lupin protein causes reduction of the LDL cholesterol in plasma and affects the LDL-to-HDL cholesterol ratio in adult patients with hypercholesterolemia after four-week supplementation. Consumption of lupin protein can reduce the cardio-vascular risk factors in patients with higher hypercholesterolemia in particular [258]. Neuromuscular electrical stimulation (NMES) accompanied by supplementation with protein-rich lupin seeds can confine the loss of the volume and strength of human calf musculature during long-term loading due to wearing a relief brace. Neuromuscular electrical stimulation combined with supplementation with lupin protein could control muscle atrophy to a large extent [259].

Many other clinical studies have also been conducted, their findings not published though. They examined the influence of consumption of lupin seeds from brine on lipid and glucose levels, on inflammatory and oxidative stress, and on the gut microbiota profile and (epi)genetic markers in healthy volunteers [260]. The effect of supplementation with lupin protein on the metabolism of glucose and insulin in the whole organism was compared with a control [261]. The influence on health (analyzing blood markers) of consumption of a liquid produced from lupin protein hydrolysates was estimated [262]. The bioaccessibility of protein and amino acids from a lupin among others was determined [263]. The efficiency of the activity of fiber from the seeds of lupin, for instance, was assessed in the context of prevention of alimentary canal and cardio-vascular system diseases [264]. The effect of lupin consumption on allergy in children was evaluated [265]. Also, the incidence of allergy [266] in healthy and atypic persons was determined [266,267], as well as allergic proteins [268] and cross reactions between the fenugreeks and other fabacean plants, including lupins, were identified [269]. The bioaccessibility of proteins and amino acids from plant-based drinks, also from lupins, was tested [270]. Among others, lupins were studied with the aim to estimate the satiety, postprandial effects, bioaccessibility of metabolites and metabolism of particular proteins in order to assess the possible results of partial replacement of meat with lupin proteins incorporated into a diet [271]. The effect of lupin consumption on losing weight (metabolic markers), on profiles of the immunity system cells, on the characteristics of adipocytes and their interaction with other cells [272], as well as on blood pressure in humans [273] and also on glycemic reactions, was evaluated [274]. A mixture of plant proteins, including ones from a lupin species, was tested against the postprandial evolution of protein metabolism [275]. The effect of a diet based on leguminous plants, including a lupin, on the clinical traits, as well as biochemical and hormonal parameters, associated with polycystic ovaries syndrome was investigated [276]. Numerous studies involving lupin species point to a wide interest in the possible influence of these plants on human health.

### 4.3. Research into the Effects of Narrow-Leaved Lupin

**Figure 13:**
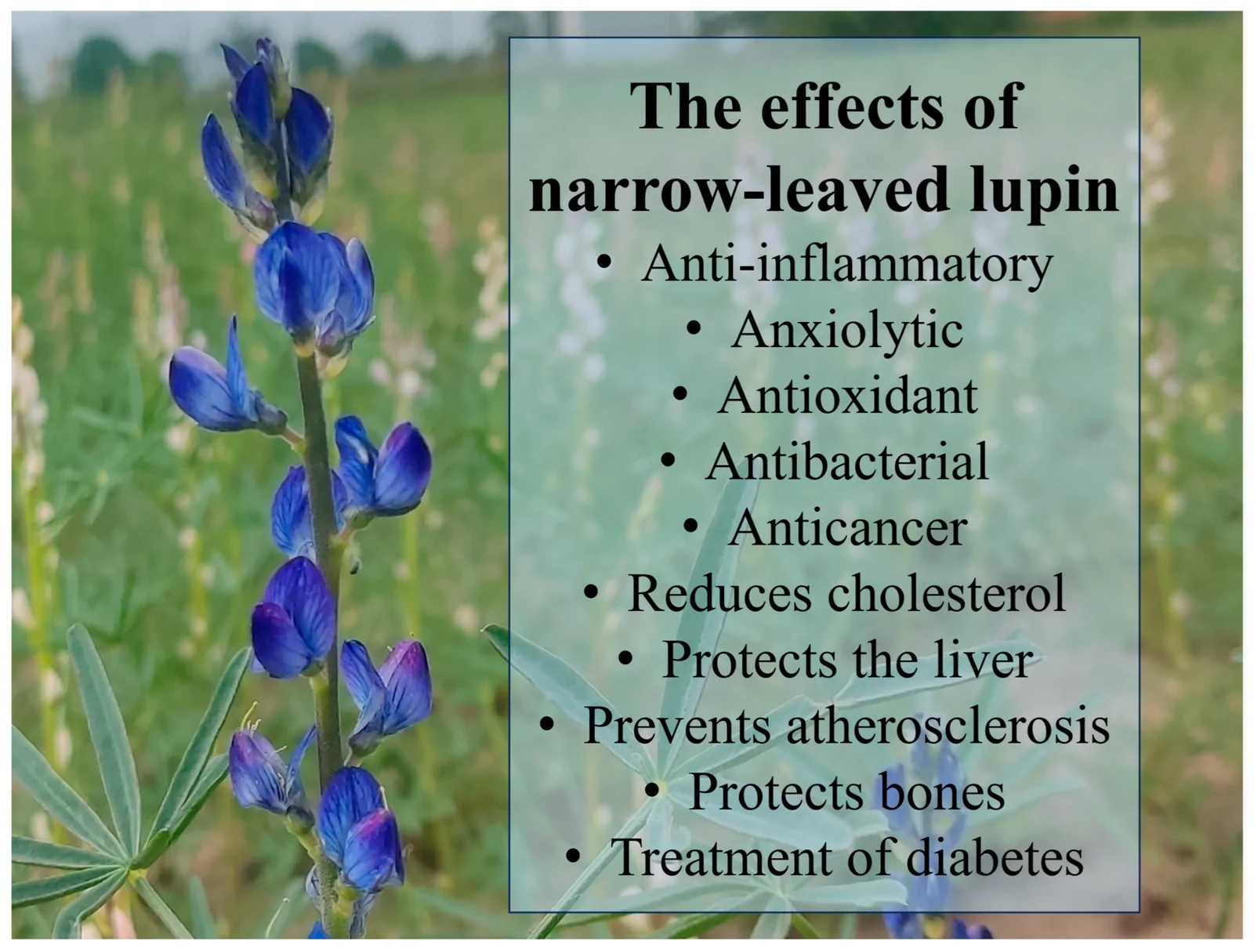
Medicinal properties of *Lupinus angustifolius*.

Many scientific studies on narrow-leaved lupin (Table 13) indicate effects in the areas shown in Figure 13: anti-inflammatory, anxiolytic, antioxidant, antibacterial, reduces cholesterol, protects the liver, prevents atherosclerosis, protects bones, treatment of diabetes and a source of anticancer substances and substances that protect healthy cells.

Studies performed on humans indicate anti-inflammatory, antioxidant, cholesterol-lowering, and antidiabetic effects, as well as health-promoting effects. Animal studies indicate anxiolytic, anti-inflammatory, antioxidant, hepatoprotective, anti-atherosclerotic, and health-promoting effects. Meanwhile, in vitro studies have shown antioxidant, anti-inflammatory, anticancer, antibacterial, and antidiabetic effects, as well as the ability to reduce osteoclastogenesis.

Thanks to these properties, narrow-leaved lupin has great potential in therapeutic nutrition. One of the most interesting compounds found in narrow-leaved lupin is β-conglutinins, which has been shown in studies to possess, amongst other things, anticancer activity. One promising avenue for the future could be to conduct more research on other types of healthy and cancerous cells, as well as to move on to human trials. From another perspective, a good approach is to identify the variety with the highest content of this valuable component and to undertake breeding research aimed at developing the best varieties for isolating this component.

As mentioned earlier, lutein is found in very high quantities in narrow-leaved lupin compared to other legumes. Lutein is known, amongst other things, for its neuroprotective, ophthalmological, eye-protecting and antidiabetic effects [277,278,279,280]. A promising avenue for future research into this substance is the selection of suitable varieties containing the highest levels of this compound, as well as the isolation and use of lutein from narrow-leaved lupin in clinical trials. We have not found any direct studies examining the health effects of lutein isolated from narrow-leaved lupin.

It would be worthwhile to conduct research on other varieties of narrow-leaved lupin in order to identify the best genotypes for clinical trials and therapeutic applications. One avenue for future research could be an attempt to identify specific compounds or a composition that may be directly responsible for all these properties. The results of such research would enable targeted breeding and allow other studies to focus on optimizing the production of higher-quality crops.

**Table 13:** Medicinal properties of *Lupinus angustifolius*.

Compounds	Effect	Methods	Reference
Protein hydrolysate	Anxiolytic	Mice ApoE^−/−^	[29]
Protein hydrolates	Antioxidant, anti-inflammatory	Human peripheral blood mononuclear cells	[281]
Drinks containing protein hydrolysates	Increased anti-inflammatory response, improved cellular antioxidant capacity Reduces the low-density lipoprotein-cholesterol (LDL-C)/high-density lipoprotein-cholesterol (HDL-C) atherogenic index and reduce total cholesterol levels	Humans 18–50 years	[282]
Protein hydrolates	Increases the liver’s antioxidant capacity and reduces inflammation; Reduces abdominal adiposity; Helps prevent the development of fatty liver disease; Ameliorates the generation of a steatotic liver	Mice ApoE^−/−^	[283]
Protein hydrolates	Prevents the early stages of atherosclerosis	Mice ApoE^−/−^	[284]
Protein hydrolysates	Anti-inflammatory	THP-1 monocytes	[35]
Protein hydrolysates	Reduces osteoclastogenesis	Peripheral blood mononuclear cells	[285]
Protein hydrolysate	Hypocholesterolemic effect	Mice ApoE^−/−^	[286]
Peptide from protein hydrolysate	Prevents inflammation	BV-2 microglial cells and mice with obesity	[287]
β-conglutin protein isoform	Prevention and treatment of diabetes and potential as anti-inflammatory	Human	[288]
Peptides	Hypocholesterolemic	Human hepatic cells HepG2	[289]
Extract from germinating and non-germinating seeds	Limited therapeutic potential	*Staphylococcus aureus*, *Pseudomonas aeruginosa*, *Eschericha coli*, *Klebsiella pneumoniae*, *Staphylococcus epidermis*	[290]
Extract from germinating and non-germinating seeds, extracts from pods; activity dependent on free phenolic acids	Limited therapeutic potential, antibacterial	SKH-1 female mice	[290,291]
		Human cancer cell lines: breast cancers, from ovarian and cervical malignancies, respectively	
		*Escherichia coli*, *Bacillus subtilis*	
Alkaloid extract	Antibacterial	*B. subtilis*, *S. aureus* and *P. aeruginosa*	[136]
Extract hydrolysate	Promotes insulin secretion	Pancreatic beta-cell lines	[292]
Seeds in diet	Cholesterol-lowering effect	Pigs	[293]
Seeds in diet	Reduced serum cholesterol and lactate	Ewes	[294]
β-conglutins	Anticancer agent that protects healthy cells	Human breast cancer cell lines and healthy non-tumorigenic epithelial cell line	[255]
β-conglutins	Adjuvant in radiation therapy	-	[295]
β-conglutins	Suppressing fungal growth and aflatoxin production	*Aspergillus flavus*, *A. niger*, *A. parasiticus*	
β-conglutins	Anticancer	Colon cancer cell lines and normal fibroblast cell line	
Protein	Hypercholesterolemic effect; Reduction in LDL cholesterol, homocysteine, triacylglycerols and uric acid	Humans	[257]
Protein	Reductions of LDL-C, non-HDL-C cholesterol and proprotein convertase subtilisin/kexin type 9	Humans	[296]
Fiber	Improves colon function and has a beneficial effect on potential risk factors for colorectal cancer	Humans	[297]

### 4.4. Usefulness of Lupin in Cosmetic Industry

**Figure 14:**
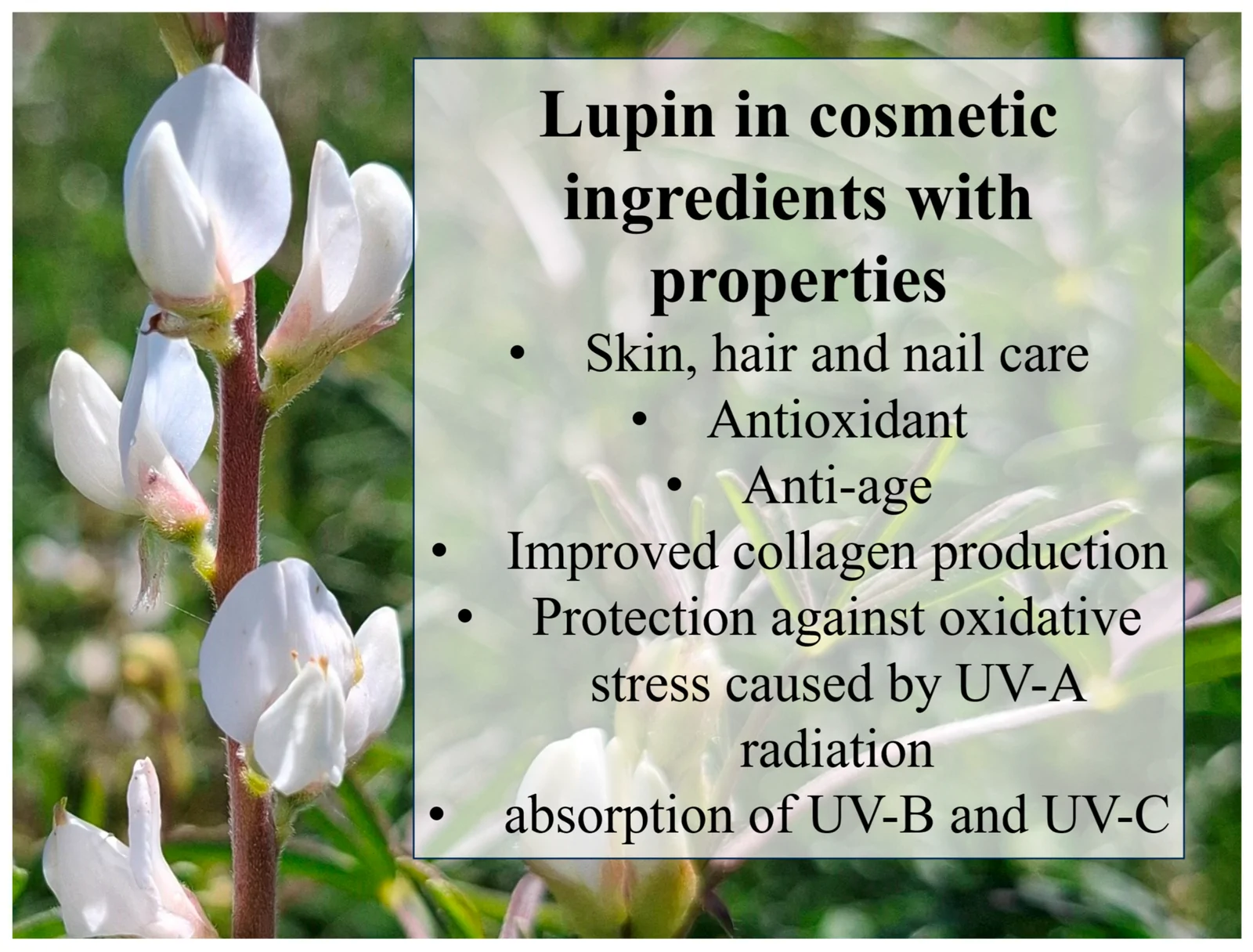
Properties of cosmetic products containing ingredients derived from lupin.

The hitherto pursued research concentrated on potential health benefits of lupin products. Current scientific research and patents point to the wide-ranging applications of cosmetic formulations containing ingredients derived from lupins (Figure 14).

Lupine amino acids: As an ingredient in cosmetics, these are obtained through the complete hydrolysis of lupin proteins. They are believed to have a nourishing effect on the hair and skin, as well as moisturizing properties [298]. Lupinus Albus Protein is recommended as a skin care ingredient due to the emollients it contains [299]. Research indicates that formulations containing lupin peptides possess antioxidant properties, offer protection against oxidative stress caused by UV-A radiation, and also stimulate collagen synthesis [300]. Lupin seed oils can be used in the production of creams containing UV filters due to their high absorption in the UV-C and UV-B ranges [301]. A cosmetic product containing lupine peptides may induce changes in the extracellular matrix of photoaged skin, which indicate a regenerative process [302]. Cellactive^®^, which contains Chlorella Vulgaris/Lupinus Albus Protein Ferment, has a beneficial effect on the skin [303]. A nanoemulgel containing lupin-derived glucosylceramide has been developed. Unpublished results from the team’s research indicate that its application to the epidermis yields promising results [304]. The cosmetic ingredient *Lupinus albus* seed oli (white lupin) and *Lupinus albus* oli unsaponifiables and the oil derived from it were assessed positively in terms of safety [305]. Lupine Amino Acids is an ingredient in preparations designed to treat skin conditions [306], for skin and hair care [307], and for the restructuring of fibers, including keratin fibers [308]. Lupinus Albus Oli Unsaponifiables were used in the treatment of damaged hair [309], as well as in hair care formulations [310]. Lupinus Albus Seed Extract and Lupinus Albus Protein are used in cosmetics for hair, skin and nail care [311], or an anti-ageing ingredient [312]. Lupinus Albus Seed Oil is a cosmetic ingredient used in the treatment of inflammation of the skin and mucous membranes, and damage caused by light exposure [313], as well as an ingredient in a UV-absorbing formula [314] and skin care formula [315]. Lupinus Subcarnosus Symbiosome Extract is used as an ingredient to inhibit protein degradation in the skin and to treat or prevent skin conditions [316], and as an ingredient in a formula designed to care for the skin and reduce the signs of photo-damage and ageing [317]. Lupinus Texensis Seed Extract is used as an ingredient in a personal care product [318,319]. Chlorella Vulgaris/Lupinus Albus Protein Ferment is used as an ingredient in a cosmetic product useful for the treatment and/or care of the skin, hair and nails [311]. Hydrolyzed Lupine Protein is used as a compound in a preparation for the treatment and/or care of the skin [320]. Furthermore, it is recommended that by-products of the lupin industry, as well as lupin itself, be used in the cosmetics industry due to the properties of the compounds they contain [321].

Making use of the generally accessible source of Cosmetics Europe (Table 14), we have compiled a list of accessible lupin components that can be found in cosmetics. These ingredients exhibit an array of properties that are beneficial for skin care, hair nourishment, scenting, nail care and skin protection, and also affect the viscosity of cosmetic products or their antistatic properties.

Ingredients coming from lupin species commonly used in the cosmetics industry are presented in Table 15. These cosmetics are dedicated for skin care as anti-wrinkle and firming creams, as well as foot and hand creams. Some of them have brightening and whitening effects on skin; eliminate freckles and pigmentation spots; show antistatic, rejuvenating and conditioning properties; benefit hair care and protect against hair loss; and also, they are components of lipsticks, scrubs and sunscreens.

A review of scientific studies highlighting the effects of lupin-derived ingredients and their health benefits, alongside the cosmetics already available on the market, indicates that the industry has a keen interest in these products. We would suggest that further research is needed on a larger number of genotypes in order to identify the best varieties for use in this sector.

### 4.5. Utility Lupin in Food Industry

**Figure 15:**
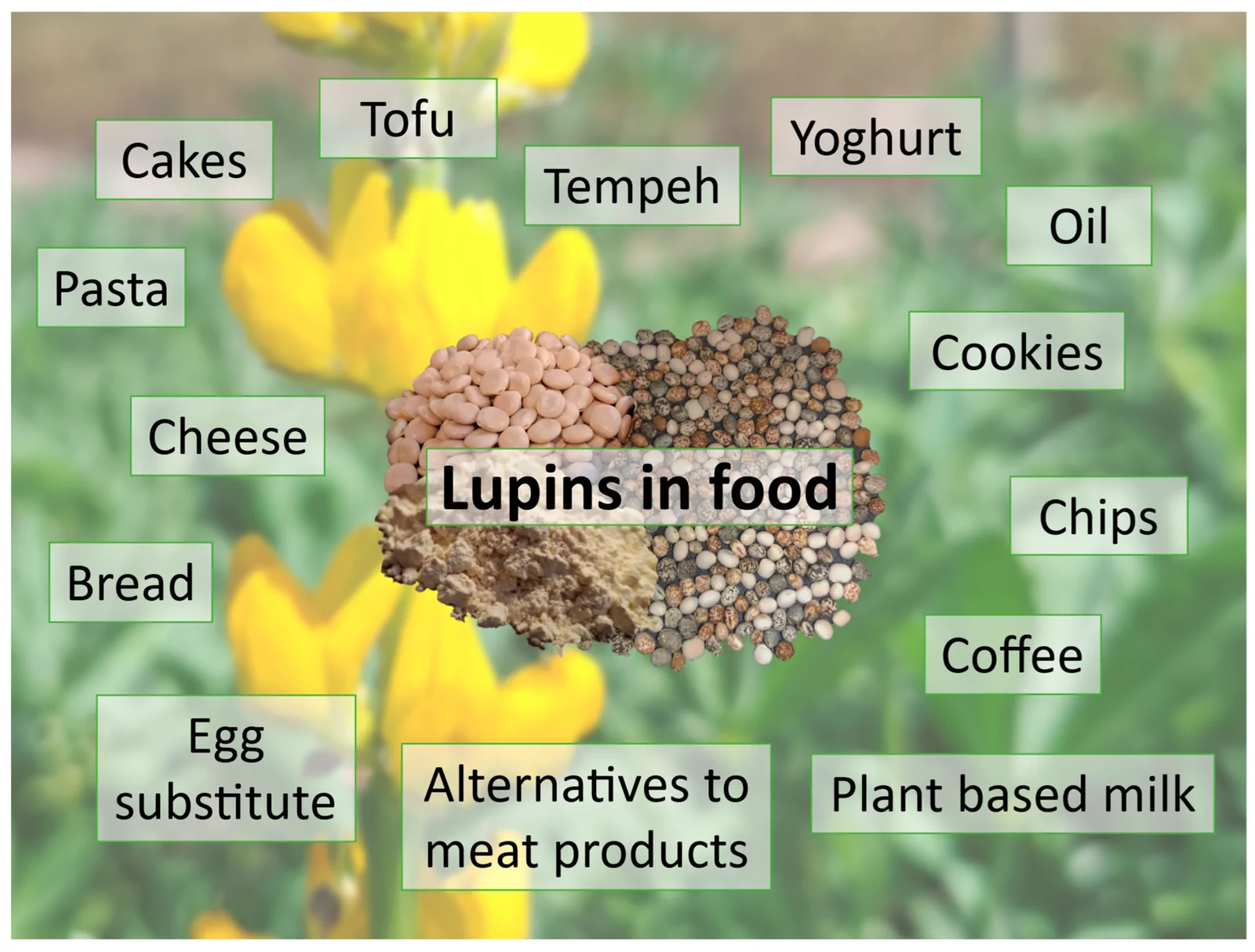
Food products made from lupin or containing lupin.

In previous instalments of this publication, biologically active and health-promoting compounds, as well as the plant’s medical potential, were highlighted. There is considerable interest amongst scientists in developing products containing lupin or made from lupin (Figure 15). Some studies suggest that products containing lupin have an unpleasant taste due to the presence of certain compounds [357]. However, some studies suggest that there is no adverse effect on sensory properties.

Research carried out on narrow-leaved lupin oil indicates its high nutritional value, stability, long shelf life and potential as a source of lipids [358]. The addition of lupin flour to biscuits does not adversely affect their sensory properties. Muffins made with lupin flour have a higher nutritional value, and the product retains its physical properties, texture and sensory qualities [359]. Muffins made with flour derived from sprouted Australian sweet lupin were characterized by higher levels of phenolic compounds and phytosterols, as well as greater antioxidant activity [360]. The addition of lupin flour to cakes is acceptable [361,362,363]. Breads containing lupin fiber did not reduce their palatability and may offer health benefits [364]; the finding regarding the taste of bread is similar in another study [365]. An egg substitute was produced for yellow cakes using lupin [366,367]. Lupin is characterized by its wide range of uses in baking [368]. Furthermore, it was used to make pasta [369,370,371]. The production of tofu containing up to 40 per cent lupin has no adverse effect on the product’s acceptability, flavor, quality or texture [372]. It was also used to make tempeh [373,374,375,376] and used to produce a milk alternative [377,378]. Other studies suggest that lupin-based ‘plant milk’ could be used in the production of frozen desserts [379] and yogurt [380,381]. The addition of lupin flour to beef sausage does not affect its acceptability [382]. Furthermore, scientific research points to the use of lupin in the production of plant-based burgers [383] and the production of meat substitutes based on lupin [383,384,385]. Research carried out indicates the potential of lupin seeds as a raw material for the development of a new group of plant-based alternatives to mature cheeses [386]. It was used to make lupin cheese as an alternative to white cheese [387]. Cheese was also made from a mixture of lupin and cow’s milk [388]. What is more, lupin was used to make A snack [389,390], lupin coffee [391], miso [392], chips [393], or lupin oil with added herbs [66].

Lupin can be found in numerous food products (produced exclusively from it or with its addition) available on the market (Table 16), among others, in the form of nutritive protein, cereals, ice creams, bakery mixtures for cookies and bread, egg substitute, meat substitute, semolina, flour, “cheeses”, “tempeh”, “tofu”, snacks from seeds in brine or roasted seeds, pastes, bars, hummus, miso, non-caffein “coffee”, “soy sauce”, cookies, bread, pasta, “butter”, crisps, lupin sprouts, “milk”, plant drinks, groats, seeds for cooking, and even in beer or other alcohols.

It is noted that it is characterized by an exceptional content of antioxidant compounds. Furthermore, the content of these compounds can be increased through germination and fermentation [452].

Scientific studies indicate the presence of undesirable flavors such as ‘beany’, ‘green’ and ‘grassy’ in lupin products. These undesirable flavors should be reduced to a level undetectable by consumers. It is noted that the reduction of unpleasant flavors in lupin products is important, and there is a need to adopt a strategy based on their elimination from food [357]. The above review and sensory analyses indicate that the addition of lupin to food or the use of lupin to create food products is acceptable to consumers. This is confirmed by other review studies that discuss the removal of non-nutritive substances by selecting sweet lupin varieties and through the appropriate preparation of the raw material for food production. The use of processes such as soaking and draining the seeds, boiling, roasting, baking and the use of microorganisms is highlighted [357]. Furthermore, sowing sweet varieties of lupin opens up the possibility of local processing into products similar to soya [357]. What can be observed from the products available on the market is shown in Table 16.

In the food industry, the situation is very similar to that in the cosmetics industry. There is considerable interest, both amongst scientists and within the industry, in the use of lupin in food products, and this offers excellent prospects for the future. There are currently a large number of products on the market made from lupin or containing lupin-derived ingredients. In order to optimize production methods and select the best genotypes in terms of quality parameters, as well as those that facilitate and streamline their use in the food industry, further research is required. The implementation of this research within the industry and close cooperation between these sectors will facilitate the use of lupin in food production. Further research and direct contact between industry members and the scientific community (in particular, researchers) can help identify the direction in which lupin breeding, and in particular, narrow-leaved lupin breeding, should be steered in the future to improve all quality parameters and optimize production.

## 5. Summary

Direct research on the narrow-leaved lupin points to the fact that the plant constitutes a source of raw materials surpassing most crop plants, as it is characterized by a unique composition of nutrients in comparison with other legumes, is a balanced source of nutritional compounds, and has protein that possesses an adequate content of amino acids and peptides, while its seeds and by-products of protein origin included into various food products enrich them with precious chemical compounds.

The most recent data obtained in the investigations pursued by the Research Centre for Cultivar Testing (COBORU) in Poland indicate that the protein content in thick-seed leguminous plants cultivated in Poland assumes the following values: the field bean *Vicia faba* 26.9–30.1%, grass pea *Lathyrus sativus* 21.7–23.8%, white lupin 34.7–37.0%, narrow-leaved lupin 28.2–30.4%, yellow lupin 41.5–44.2%, soya bean 35.7–42.2%. The fat content in particular species has been determined as follows: the white lupin 11.9–13.4%, narrow-leaved lupin 6.7–7.5%, yellow lupin 5.9–6.8%, soya bean 19.9–23.7% [453]. Comparing the content of fat and protein in meat—which is as follows: beef—protein 20.4%, fat 11.0%; pork—protein 20.1%, fat 9.0%; chicken—protein 18.9%, fat 10%; fish and sea food—protein 17.4%, fat 3.9% [454]—points to the fact that the protein and fat contents in leguminous plants substantially surpass the figures recorded for different type of meat, fish and sea food. Current recommendations of the EAT–Lancet Commission for consumption of legumes by an average person should be at the level of 75 g daily (annually 27.4 kg) [455].

Some of the most advantageous substances present in the narrow-leaved lupin seed are its unique conglutins, whose health-benefiting properties have been discussed above [18,31,32,33,34,35]. An interesting aspect in view of the *L. angustifolius* wellness-enhancing potential is also its content of lutein. As mentioned above, the lutein content in the narrow-leaved lupin reaches 35.95 mg/kg, whereas in the soya bean—from which lutein is acquired at the commercial scale—its level ranges from 1.35 mg/kg to 3.2.08 mg/kg [68]. This means an economically justifiable perspective for the narrow-leaved lupin as a new source of lutein acquisition. On the other hand, antinutritional substances in leguminous plants are commonly recognized [456]. For instance, in the green gram, chickpea, pigeon pea, lentil, black gram, field pea, kidney bean, cowpea and faba bean, enzymatic inhibitors and non-enzymatic inhibitors have been detected [456]. The soya bean contains an array of antinutritive compounds such as trypsin, chymotrypsin, lectins, antivitamins, goitrogens, cyanogenic compounds, glycinine, β-conglycinine, and phytoestrogens. The pea displays anti-trypsin activity and contains tannins. The field bean contains tannins, protease inhibitors, lectins, glycosides, phytates, alpha amylase inhibitors and oligosaccharides. *Phaseolus* L. possess a trypsin inhibitor, lectins, phytic and phenolic compounds, and oligosaccharides from the raffinose group. In the yellow and white lupins, there are alkaloids and raffinose oligosaccharides. The narrow-leaved lupin has been indicated as containing antinutritional oligosaccharides, alkaloids and phytates [457]. However, as mentioned above, breeding advances have made it possible to develop varieties with significantly reduced alkaloid content. Scientific studies report that their current content ranges from 0.0005 to 2.8752% of the dry seed weight [138]. Although the presence of higher alkaloid content in some cultivars is a fact, these are crops aimed to serve as green manure or aftercrop and are not intended for the feed or food industry. Research demonstrates that the content of phytates in lupins is comparable with the values noted for some other leguminous plants such as the chickpea or mung bean, but it is lower than in the red bean and soya bean [458]. Among *Lupinus* species, in the narrow-leaved lupin, the content of phytates is the lowest (0.58%) relative to the white (0.79%) and yellow lupin (0.96%) [459,460]. Moreover, other studies indicate that the content of phytic acid in *L. angustifolius* is low in comparison with other leguminous plants [461]. Additional advantages of the narrow-leaved lupin is its ability to adapt to ecoclimatic conditions since its vegetation season is shorter than those of other domesticated species of lupins, and its ripening shorter and more uniform, especially in non-branching cultivars of this species, which produce flowers and mature earlier and more evenly than the branching ones [462]. Some narrow-leaved lupin cultivars are less susceptible to frost damage (ground frost) than the yellow lupin, giving a better yield [463].

Making use of *L. angustifoliust* constituents maximizes the economic value of products. Including the narrow-leaved lupin products into a diet can bring about many health benefits [452,464,465]. However, all products that are an alternative to different meat and fish should comply with nutrition standards, stabilization and enrichment with certain nutrients taken into consideration [454].

A distinctive feature of this review is the combination of current scientific knowledge on the composition, nutritional value, bioactive compounds and potential applications of narrow-leaved lupin with a comprehensive analysis of commercially available lupin-based products in cosmetics and food. The products described in scientific publications have, for the most part, already been commercialized, whilst there are also products on the market for which we were unable to find any scientific references. We can conclude that the industry is keen to use lupin in various products, which may indicate market interest in lupin-based products.

## 6. Conclusions

In our opinion—taking into account a similar composition of micro- and macroelements as found in other leguminous plants; a good content of vitamins; a high content of β-conglutin; favorable composition of amino acids, health-enhancing flavonoids, phytosterols and phenolic compounds, and carotenoids; a very low content of alkaloids and high contents of protein and fat, and the quality of both; and also negligible amounts of antinutritional factors (except for oligosaccharides from the raffinose group, whose content in all fabaceans is the focus of plant breeders’ attention with respect to possible reduction)—the narrow-leaved lupin can successfully compete with soya bean, and in some aspects also with the white and yellow lupins, at least owing to the presence of β-conglutin or lutein. Considering all aspects connected with chemical compounds occurring in *L. angustifolius*, the plant can successfully compete with other lupins in the cosmetics industry and become a source of better quality with respect to ingredients that are made use of in cosmetic products.

This review pertaining to nutritional products from lupins and food products that contain the narrow-leaved lupin components demonstrates the potential for the comprehensive application of lupins and the possibilities of their use. Compared to other leguminous plants, the soya bean in particular, they can be a substitute and successfully become a competitor in vegan products, as well as with respect to versatility of use. On account of its wellness-promoting composition of compounds and beneficial effect on health, confirmed by numerous scientific studies, the narrow-leaved lupin should—in the first place—be used in the food industry.

The use of sweet varieties of narrow-leaved lupin is widespread due to their wide availability. The reduction in alkaloid levels through breeding programs was achieved by introducing low-alkaloid genotypes, which made it possible to develop sweet varieties. Sweet varieties are currently available with low alkaloid content not exceeding 0.02 or 0.01%, as well as bitter varieties containing higher levels of alkaloids [5]. Both sweet and bitter varieties are currently available [466,467,468,469].

We agree with the view that some products containing lupin may have an unpleasant aftertaste [357]; however, as we mentioned earlier, some products containing lupin or made from it do not have an unpleasant taste. The integrated approach is to take varietal differences into account, as well as the identification and subsequent selection of lupin varieties with a reduced aftertaste [357].

Given the variety of lupin products available on the market, as well as their large number, it can be concluded that there is interest in this type of product. Furthermore, the products launched by companies must be appealing to consumers in terms of their sensory properties. If the products were not acceptable, they would not be produced and would not be available for purchase.

One way of promoting lupin products or products containing lupin should be to raise public awareness of the possibility of consuming lupin, to highlight the health benefits associated with consuming such products, and to disseminate scientific knowledge concerning this group of plants.

The most important findings arising from this literature review are the need to investigate a greater number of narrow-leaved lupin varieties and genotypes, as well as these lupins, in terms of composition, health-promoting properties, quality and utility parameters, with a view to subsequently selecting the best ones for use in medicine, the food industry or the cosmetics sector. The results of such research would enable breeders to explore new avenues for developing varieties, not only in terms of yield-related traits, resistance, and the protein content in the pulp or fiber, but also in terms of quality parameters, with the aim of increasing the most health-promoting and unique compounds found in narrow-leaved lupin, whilst also reducing or completely eliminating other components that limit its application.

This review as a whole points to the enormous potential for the use of narrow-leaved lupin. In particular, there is a need to prioritize cooperation and a joint approach among plant breeders, scientists and the industry. A shared research agenda and collaboration could contribute to the development of narrow-leaved lupin varieties for specific and unique applications in medicine, cosmetics and the food industry. Conventional breeding—the creation of new varieties—is a very lengthy process. The use of more advanced biotech breeding methods, such as new gene-editing techniques, the use of molecular markers in selection, or the application of OMICS technologies, would enable the rapid development of narrow-leaved lupin applications.

## 7. Challenges and Future Perspectives

Taking into account the entire review, certain conclusions and challenges arise regarding the future use of narrow-leaved lupin:

- Identifying genotypes that contain the highest levels of valuable components such as β-conglutin or lutein, which will enable them to be extracted more efficiently;
- Typing genotypes with the lowest levels of undesirable substances, such as oligosaccharides from the raffinose group, with a view to undertaking breeding programs aimed at developing varieties with reduced levels of such compounds;
- Undertaking further clinical trials;
- Optimizing methods for extracting substances useful in medicine on a larger scale;
- Increasing the production of lupin-based products, whilst taking into account safety requirements relating to allergens;
- Testing and selecting varieties that meet the relevant criteria for various industries, such as the food industry (tempeh or coffee) or the cosmetics and pharmaceutical industries;
- Standardizing standardized seed lots of varieties that meet the relevant performance requirements;
- Determining which varieties are best suited to the production of foods such as tempeh or coffee, which may have different requirements in terms of seed characteristics and the sourcing of ingredients for cosmetics;
- Ensuring the safety of plant-based protein;
- Close cooperation among plant breeders, scientists and the industry.

## Figures and Tables

**Table 1:** The content of lectins and amino acids in *Lupinus angustifolius* seeds.

Chemical Compound		Content	Source
Proteins			[28]
Conglutins	α-Conglutin	24%	
	β-Conglutin	56%	
	δ-Conglutin	15%	
	γ-Conglutin	6%	
Amino acids			
Aspartic acid (Aspartic acid + asparagine)		11.6 g/100 g 9.5 g/16 gN	[36,37]
Glutamic acid (Glutamic acid + glutamine)		25.6 g/100 g 21.1 g/16 gN	
Serine		7.1 g/100 g 4.5 g/16 gN	
Histidine		2.7 g/100 g 2.7 g/16 gN	
Glycine		4.6 g/100 g 4.2 g/16 gN	
Threonine		4.9 g/100 g 3.5 g/16 gN	
Arginine		11.5 g/100 g 10.1 g/16 gN	
Alanine		3.8 g/100 g 3.4 g/16 gN	
Proline		4.6 g/100 g 3.7 g/16 gN	
Tyrosine		5.9 g/100 g 3.8 g/16 gN	
Valine		3.9 g/100 g 4.2 g/16 gN	
Methionine		1.3 g/100 g 0.7 g/16 gN	
Cysteine		3.5 g/100 g 1.5 g/16 gN	
Isoleucine		5.5 g/100 g 4.3 g/16 gN	
Leucine		8.7 g/100 g 7.0 g/16 gN	
Phenylalanine		5.2 g/100 g 4.0 g/16 gN	
Lysine		5.4 g/100 g 4.8 g/16 gN	
Tryptophan		0.6 g/100 g 3.5 g/16 gN	

**Table 2:** Fat and fatty acids in *Lupinus angustifolius*.

Part of Narrow-Leaved Lupin	Chemical Compound		Content		Source
Fatty acids					
Seed	Glycerophospholipids	1.2 Dilinoleoyl-PC	0.01–0.05 mg/kg		[49,50,51]
		1.2 Dioleoyl-GLP	6.13–8.57 mg/kg		
	Glycerolipids	Liinoleoyl-rac-GL	58.09–88.80 mg/kg		
		Loleoyl-rac-GL	2.43–5.96 mg/kg		
	Sterols	Stigmasterol	2.04–2.93 mg/kg		
		Campasterol	2.13–3.05 mg/kg		
	Unsaturated fatty acids	Oleic acid + cis-vaccenic	196.04–282.70 mg/kg		
		Ethyloleate	0.03–0.35 mg/kg		
		Linoleic acid	331.71–423.32 mg/kg		
		Ethyl-linoate	0.64–1.32 mg/kg		
		Methyl-linoate	0.99–1.27 mg/kg		
		Linolenic acid	40.13–52.28 mg/kg		
		Miristoleic acid	30.21–54.21 mg/kg		
		Palmitoleic acid	19.20–25.92 mg/kg		
		Erucic acid	30.21–54.21 mg/kg		
	Saturated fatty acids	Palmitic acid	123.68–182.97 mg/kg	11.4–12.0%	
		Stearic acid	61.40–96.35 mg/kg	6.5–8.2%	
		Arachidic acid	4.40–8.10 mg/kg	1.0%	
		Behenic acid	4.51–8.08 mg/kg	2.0%	
		Lignoceric acid	2.11–3.24 mg/kg	0.6%	
		Myristic acid	1.93–3.26 mg/kg		
		Margaric acid	3.50–5.19 mg/kg	0.3%	
		C18:1n-9 (Oleic acid)	30.8–36.2%		
		C18:1n-7 (Vaccenic acid)	0.8%		
		C20:1 (Gadoleic acid)	0.3%		
		C18:2n-6 (Linoleic acid)	37.8%		
		C18:3n-3 (Linolenic acid)	5.1%		
		Cinoleic acid C18:2	41.7–38.3%		
		Arachidic acid C20:0	0.9–1.2%		
		α-Linolenic acid C18:3	4.9–6.4%		
		Behenic acid C22:0	1.8–2.3%		
		Lignoceric acid + eicosapentaenoic acid C24:0 + C20:5	0.4–0.5%		
Polyunsaturated					
Seed oil	SAFA, saturated fatty acids		19.02–21.7%		[52]
	MUFA, monounsaturated fatty 24 acids		37.49–47.29%		
	PUFA, polyunsaturated fatty acids		31.01–44.64%		
	UFA/SFA, the ratio of unsaturated to saturated fatty acids		3.61–4.26%		
Triacylglycerols					
Seed oil	LnLnL		0.03%		
	LnLL		0.29–0.69%		
	LnLO		1.37–6.51%		
	LLL		0.83–1.59%		
	LnLP		0.45–1.00%		
	LLO		9.37–23.66%		
	LnOO		4.06–8.70%		
	LnOP		0.22–0.58%		
	LOO		23.22–25.82%		
	LLS		12.44–15.88%		
	LPP		0.62–1.12%		
	OOO + LnSS		8.28–19.86%		
	LOS		3.77–7.37%		
	OOP		3.43–9.37%		
	LSP		0.32–0.78%		
	POP		0.31–0.54%		
	LSS		0.20–0.47%		
	OOS		0.21–0.28%		
	POS		1.30–6.64%		
	SPP		0.10–0.31%		

The fatty acid residues present in triacylglycerol: Ln—linolenic acid, L—linoleic acid, O—oleic acid, P—palmitic acid, S—stearic acid.

**Table 3:** Carotenoids in *Lupinus angustifolius*.

Part of Narrow-Leaved Lupin	Chemical Compound	Content	Source
Carotenoids			[66]
Oil	Neoxanthin equival	5.54 µg/mL	
	(all-E)-Lteoxanthin A	1.09 µg/mL	
	(all-E)-Luteoxanthin B	2.89 µg/mL	
	(9Z)-Violaxanthin	1.47 µg/mL	
	(all-E)-Lutein	74.2 µg/mL	
	9-Cislutein	3.59 µg/mL	
	7-Cislutein	2.77 µg/mL	
	Zeaxanthin	9.73 µg/mL	
	5,6-Epoxyβcarotene	5.20 µg/mL	
	β-Carotene	53.9 µg/mL	
	9-Cis-β-carotene	10.2 µg/mL	
Seed	Violaxanthin	0.32–3.44 mg/kg	[52,67]
	Violaxanthin	0.32–3.44 mg/kg	
	Lutein	11.17–35.95 mg/kg	
	Zeaxantin	4.9–16.7 mg/kg	
	α-Carotene	0.35–3.03 mg/kg	
	9-Z-β-carotene	0.29–1.86 mg/kg	
	13-Z-β-Carotene	0.25 mg/kg	
	β-Carotene	0.5 mg/100 g	
		0.17–1.29 mg/kg	

**Table 4:** Phytosterols in *Lupinus angustifolius*.

Part of Narrow-Leaved Lupin	Chemical Compound	Content	Source
Phytosterols			
Seed	Ergosterol	2.77 µg/g	[100]
Oil	Campesterol	2031.0–2653.4 mg/kg	[52]
	Campestanol	79.5–231.5 mg/kg	
	Stigmasterol	568.7–788.1 mg/kg	
	β-Sitosterol	4398.1–5714.5 mg/kg	
	Sitostanol	60.0–93.2 mg/kg	
	Δ5-Avenasterol	193.2–762.7 mg/kg	
	α-Amyrin (5,24-stigmastadienol)	102.4–167.1 mg/kg	
	Δ7-Stigmastenol	46.4–83.2 mg/kg	
	24-Methylenecycloartanol	5612.2–6966.0 mg/kg	

**Table 5:** Fiber and sugars in *Lupinus angustifolius*.

Chemical Compound		Content	Source
Fiber and sugars			[107]
Cell wall components	Non-starch polysaccharides	401–407 g/kg	
	Neutral detergent fiber	252–269 g/kg	
	Acid detergent fiber	212–222 g/kg	
	Crude fiber	151–166 g/kg	
	Acid detergent lignin	20–23 g/kg	
	Hemicellulose	40–47 g/kg	
	Cellulose	195–193 g/kg	
Oligosaccharides	Sucrose	14.0–19.2 g/kg	[107,108]
	Galactopinitol	1.4–1.8 g/kg	
	Galactoinositol	1.3–1.6 g/kg	
	Raffinose	5.32–20.20 g/kg	
	Digalactopinitol	2.6–3.9 g/kg	
	Digalactoinositol	3.4–3.7 g/kg	
	Stachyose	29.9–75.38 g/kg	
	Verbascose	7.1–24.59 g/kg	
	Ajugose	16.7–26.2 µmol/g	[109]
Non-starch polysaccharides	Rhamnos	3.0–4.0 g/kg	[107]
	Fucose	1.0–2.0 g/kg	
	Arabinose	41.0–45.0 g/kg	
	Xylose	31.0–33.0 g/kg	
	Mannose	7.0–8.0 g/kg	
	Galactose	149.0–150.0 g/kg	
	Glucose	123.5–123.3 g/kg	
	Uronic acids	43.0–45.0 g/kg	

**Table 6:** Alkaloids in *Lupinus angustifolius*.

Part of Narrow-Leaved Lupin	Chemical Compound	Content	Source
Alkaloids			[137]
Seed	Gramine	11.0–0.0 mg/kg	
	Ammodendrine	2.6 mg/kg	
	Lupinine	12.7 mg/kg	
	Angustifoline	0.0–265.0 mg/kg	
	Sparteine	0.0–3.29 mg/kg	
	Lupanine	8.99–79.3 mg/kg	
	13-α-Hydroxylupanine	13.3–101.0 mg/kg	
	13-Tigloyloxylupanine	40.1–50.5%	[135]
	Lupanine	15.2–43%	
	11,12-Dehydrosparteina	12.3%	
	Tetrahydrorhombifolina	10.5%	
	Sparteine	0.015%	
	Ammodendrine	0.014%	
	α-Isolupanine	0.154%	
Green parts, above-ground parts	Ammodendrine	1.10%	[136]
	Isoangustifoline	0.78%	
	Tetrahydrorhombifoline	1.14%	
	Angustifoline	2.94%	
	α-Isolupanine	1.67%	
	5,6-Dehydrolupanine		
	Lupanine	23.55%	
	11,12-Dehydrolupanine		
	13α-Hydroxylupanine	50.78%	
	13α-Acetoxylupanine	1.54%	
	13α-Isovaleroyloxylupanine	1.13%	
	13α-Valeroyloxylupanine	1.65%	
	13α-Tigloyloxylupanine	6.77%	
	13α-cis-Cinnamoyloxylupanine	2.60%	
	13α-cis-Cinnamoyloxy-17-oxolupanine		

**Table 7:** Vitamin content in the seeds of *L. angustifolius* [52,67,108,156].

Chemical Compound		Content
Vitamins		
Vitamin B1		5.66–7.12 mg/kg
Vitamin B2		2.36–2.74 mg/kg
Vitamin E	α-Tocopherol	1.42–8.61 mg/kg
	β-Tocopherol	2.23–3.12 mg/kg
	γ-Tocopherol	10.30–91.52 mg/kg
	δ-Tocopherol	1.26–1.41 mg/kg

**Table 8:** Macroelements and microelements in *L. angustifolius* [49].

Chemical Compound		Content
Microelements	Na	1.01–1.19 mg/g
	Mg	2.00–2.45 mg/g
	K	12.63–13.61 mg/g
	Ca	1.46–2.53 mg/g
Macroelements	Mn	28.13–123.23 µg/g
	Fe	52.54–64.27 µg/g
	Zn	5.10–6.46 µg/g
	Cu	5.10–6.46 µg/g
	Ni	1.25–1.61 µg/g
	Cr	0.91–1.81 µg/g
	Se	0.09–0.16 µg/g
	Co	0.03–0.18 µg/g
	Sr	9.09–28.49 µg/g
	Al	0.94–6.27 µg/g
	Pb	0.06–0.11 µg/g
	Cd	0.03–0.05 µg/g
	As	0.02–0.03 µg/g
	Ag	<0.005 µg/g

**Table 9:** Flavonoids found in *Lupinus angustifolius*.

Part of Narrow-Leaved Lupin	Chemical Compound			Content			Source
Flavonoids and their glycosides							
Roots	Genistein						[181]
	2′-Hydroxy genistein						
	3′-Omethylorobol						
	Wighteone						
	Luteone						
	Licoisoflovone A						
	Lupisoflovoce						
	Lupalbigenin						
	Hydroxylupalbigenin						
	Alpinumisoflavone						
	Parvisoflavone B						
	Angustone c						
	Chandalone						
	Licoisoflavone B						
	Angustone B						
	Isoderrone						
	Isochandalone						
	2′-Hydroxygenistein						
	4′,7-O-Diglucoside						
	Genistein 4′-O-glucoside-7-O-(6″-O-malonyl) glucoside						
	2′-Hydroxygenistein 7-O-(6″-O-malonyl) glucoside						
	Genistein 4′-O-glucoside						
	Genistein 4′-O-(6″-O-malonyl) glucoside						
Leaves	Apigenin 7-O-xylosylglucoside						
	3′ or 4′-Methylluteolin 7-O-xylosylglucoside						
	3′ or 4′-Methylluteolin 7-O-glucoside						
	3′ or 4′-Methylluteolin 7-O-xylosylglucoside						
	Malonylated; 3′ or 4′-methylluteolin 7-O-(6″-O-malonyl) glucoside						
	3′ or 4′-Methylluteolin						
Leaves and roots	Genistein 4′,7-O-diglucoside; 2′-hydroxygenistein 7-O-glucoside						
	Genistein 6 or 8-C-glucoside						
	Genistein 7-O-glucoside						
	Genistein 6 or 8-C-(6″-O-malonyl) glucoside						
	Genistein 7-O-(6″-O-malonyl) glucoside						
	2′-Hydroxygenistein						
	Genisteina						
	Luteone						
	Wighteone						
Dihydroflavonols and flavones							
Seed	Dihydroquercetin derivative						[183]
	Dihydrokaempferol acetylglycoside						
	Apigenin 7-apioglucoside						
	Apigenin derivative						
	Apigenin 7-neohesperidoside						
	Luteolin derivative						
	Diosmetin rutinoside						
	Diosmetin neohesperidosido						
	isoflavones						
	Genistein derivative						[182]
	Genistein glucoside						
	Genistein acetylglycoside						
	Flavone		Apiin		0.12–0.19 mg/kg	[50]	
			Luteolin		1.23–2.31 mg/kg		
			Hesperidin		0.19–0.29 mg/kg		
			Apigenin-7-glc		0.17–0.25 mg/kg		
	Flavanone		Naringenin		0.03–0.05 mg/kg		
	Flavonols		Isorhamnetin-3-rutinoside		0.00–0.09 mg/kg		
	Flavonoids		Apigenin heteroside		665.9–692.0 mg/kg		[184,185]
Seed coat	Flavones		Apigenin-7-O-glucoside (apigenin-7-O-β-glucopyranoside (as vitexin equivalent))		24.17–83.14 μg/g		[186]
			Apigenin-7-O-β-apiofuranosyl-6,8-di-C-glucopyranoside (as vitexin equivalent)		697.85–1011.82 μg/g		
			Vicenin 2		24.65–59.53 μg/g		
			Apigenin-7-O-β-glucopyranoside (as vitexin equivalent)		22.42–91.67 μg/g		
	Isoflavones		Genistein		22.30–62.60 μg/g		
	Dihydroflavonols		Aromadendrin-6-C-β-D-glucopyranosyl-7-O-[β-D-apiofuranosyl-(1→2)]-O-β-D-glucopyranoside glucopyranoside (as taxifolin equivalent)		34.20–61.03 μg/g		
9-Day sprouts	Dihydrokaempferol acetylglycoside						[182]
	Apigenin derivative						
	Apigenin 7-neohesperidoside						
	Luteolin 7-glucoside						
	Luteolin derivative						
	Luteolin glucopyranosyl acetate						
	Diosmetin rutinoside						
	Diosmetin neohesperidosido						
	Diosmetin derivative						
	Diosmetin apiofuransyl acetylglucoside						
	Eriodictyol derivative						
	Genistein derivative						
	Genistein glucoside						
	Genistein apiofuranosyl diglycoside						
	Genistein apiofuranosyl glycoside						
	Genistein acetylglycoside						
	Genistein acetyldiglycoside						
	Genistein glucoside (genistin)						
	Hidroxygenistein-7 glucoside						
	Hidroxygenistein-7 acetylglucoside						
	Genistein						

**Table 10:** Phenolic compounds found in *Lupinus angustifolius*.

Part of Narrow-Leaved Lupin	Chemical Compound		Content	Source
Phenolic compounds				[50]
Seed	Benzoic acid	Anthranilic acid	0.031–0.035 mg/kg	
		Vanillin	0.01–0.02 mg/kg	
		Vanillic acid	0.31–0.40 mg/kg	
	Phenylpropanoids	p-Coumaric acid	0.46–0.62 mg/kg	
		Caffeic acid	0.00–0.11 mg/kg	
		Ferulic acid	1.10–1.47 mg/kg	
		Catechol	3.20–4.45 mg/kg	
	Hydroxybenzoic and hydroxycinnamic compounds	Protocatechuic acid glucoside		[182]
		Protocatechuic acid		
		p-Hydroxybenzoic acid		
		Hydroxybenzoic acid derivative		
		p-Hydroxybenzoic aldehyde		
		p-Hydroxyphenyl acetic acid		
		p-Vanillin		
		trans p-Coumaroyl glutaric acid		
		trans Ferulic acid		
		Coniferaldehyde		
Seed coat	Hydroxycinnamics	Cinnamic acid glucoside (as trans-cinnamic acid equivalent)	17.10–31.40 μg/g	[186]
		Dicaffeoylquinic acid (as caffeic acid equivalent)	42.31–89.03 μg/g	
	Protocatechuic acid		5.68–63.38 μg/g	
	p-Coumaric acid glucoside (p-coumaric acid equivalent)		3.13–7.09 μg/g	
	Ferulic acid glucoside as ferulic acid equivalent		7.36–9.41 μg/g	
	Ferulic acid		5.63–9.15 μg/g	
9-Day sprouts	Hydroxybenzoic and hydroxycinnamic compounds	Protocatechuic acid glucoside		[182]
	Hydroxybenzoic acid derivative			
	p-Hydroxyphenyl acetic acid			
	p-Vanillin			
	trans p-Coumaroyl glutaric acid			
	trans p-Coumaric acid			
	trans Ferulic acid glycoside			
	trans Feruloyl malic acid			
	trans Ferulic acid			

**Table 11:** Volatile organic compounds and terpenoids in *Lupinus angustifolius*.

Part of Narrow-Leaved Lupin	Chemical Compound	Content	Source
Volatile compounds			
Seed	Lupeol	29.58–87.66 mg/kg	[50]
	Acetic acid	37.8%	[66]
	Pentanol	3.47%	
	3-(E)-hexen-1-ol	0.64%	
	hexanol	28.1%	
	benzyl alcohol	1.17%	
	Hexanal	3.10%	
	γ-Butalactone	4.66%	
	2-Pentylfuran	14.0%	
	α-Thujene	2.81%	
	p-Cymene	0.62%	
	α-Phellandrene	3.32%	
	Limonene	0.30%	
Seed/lupin flour	Oct-1-en-3-one		[221]
	2-Acetyl-1-pyrroline		
	(Z)-Octa-1,5-dien-3-one		
	3-Isopropyl-2-methoxypyrazine		
	Acetic acid		
	(Z)-Non-2-enal		
	3-Isobutyl-2-methoxypyrazine		
	(E)-Non-2-enal		
	(E, Z)-Nona-2,6-dienal		
	2-Methylbutanoic acid/3-methylbutanoic acid		
	Pentanoic acid		
	γ-Octalactone		
	(E, E, Z)-Nona-2,4,6-trienal		
	4-(2,6,6-Trimethyl-1-cyclohexenyl)-3-buten-2-one (β-Ionone)		
	3-Hydroxy-2-methyl-pyran-4-on (maltol)		
	trans-4,5-Epoxy-(E)-dec-2-enal		
	γ-Nonalctone		
	γ-Decalactone		
	3-Hydroxy-4,5-dimethyl-2(5H)-furanone (sotolone)		
	Ethyl vanillin		
	Vanillin		
	Phenylacetic acid		
Seed	Butanoic Acid, 2-Methyl-Ester/Butyric acid	0.03–0.08%	[223]
	P-Xylene	0.0–0.02%	
	Tricylene	0.02–0.03%	
	α-Thujene	1.13–2.34%	
	α-Pinene	1.16–1.54%	
	Bicyclo(3.1.0)Hex-2-Ene, 4-Methylene-1-/Beta-thujene	0.07–0.11%	
	Bicyclo(2.2.1)Heptane,2,2-Dimethyl-3-Camphene	0.0–0.85%	
	Sabinene	0.11–0.16%	
	β-Pinene	0.36–0.47%	
	1-Octen-3-One	0.00–0.09%	
	1-Octen-3-Ol	0.11–0.25%	
	3-Octanone	0.53–0.67%	
	Geranyl Formate	0.00–3.28%	
	Ethyl Amyl Carbinol	0.0–0.5%	
	1-Phellandrene	0.49–0.62%	
	1,3,6-Octatriene, 3,7-Dimethyl-, (E)-	0.04–6.80%	
	δ 3-Carene	0.19–0.20%	
	α-Terpinene	0.0–2.99%	
	2-Propen-1-Ol, 3-Phenyl-	0.0–16.08%	
	D-Limonene	2.76–3.42%	
	Eucalyptol (1,8-Cıneole)	0.73–0.87%	
	3-Octen-5-Yne, 2,7-Dimethyl-, (E)	0.0–6.17%	
	Santolina Triene	0.0–6.16%	
	M-Mentha-4,8,Diene-(1S, 3S)	11.12–15.70%	
	Trans Sabinene Hydrate	0.17–0.43%	
	Alpha-Methyl-Alpha-(4-Methyl-3-Pente)	0.0–0.22%	
	α-Terpinolene	0.35–0.59%	
	Cyclohexanol, 2-Methyl-5-(1-Methylethe	0.0–19.37%	
	2-Cyclohexen-1ol, 1-Methyl-4-(1-Methyl	0.0–0.07%	
	Alloocimene	0.44–0.47%	
	Cis-Epoxy-Ocımene	0.0–0.09%	
	2-Cyclohexen-1-Ol, 1-Methyl-4-(1-Methyl	0.0–0.05%	
	2,4,6-Octatriene,2,6-Dimethyl-, (E, Z)	0.19–0.20%	
	Cis-3-Hexenyl İso-Butyrate	0.0–0.10%	
	Cyclohexanone, 5-Methyl-2-(1-Methylethe-(Cis)	1.97–2.12%	
	Cyclohexanone, 5-Methyl-2(1-Methylethe (Trans)	0.0–0.64%	
	Bicyclo(2.2.1)Heptan-2-Ol, 1,7,7-Trimethyl	0.0–0.83%	
	İsopulegone	0.14–0.17%	
	3-Cyclohexen-1-Ol, 4-Methyl-1-(1-Methyl	0.0–0.64%	
	Benzenemethanol, 4-(1-Methylethyl)-	0.0–0.03%	
	Cis 3 Hexenyl Butyrate	0.0–0.04%	
	3-Cyclohexen-1-Methanol, α, α	0.0–0.11%	
	Dihydrocarvone	0.0–0.25%	
	Cyclohexanone, 2-Methyl-5-(1-Methylethe-	0.0–0.17%	
	Z-3-Hexenyl 2-Methylbutanoate	0.0–0.17%	
	Thyml Methyl Ether	0.0–2.82%	
	Thymoquinone	0.0–1.94%	
	Phenol, 5-Methyl-2-(1-Methylethyl)-	0.07–0.27%	
	Alpha-Terpinyl Propionate	0.0–0.03%	
	Phenol, 2-Methyl-5-(1-Methylethyl)-	0.0–3.0%	
	Carbofurane	0.0–0.02%	
	Copaene	0.0–0.05%	
	(−)-β Bourbonene	0.0–0.04%	
	Caryophyllene	0.0–0.40%	
	(+)-Aromadendrene	0.0–0.11%	
	γ-Muurolene	0.0–0.03%	
	Germacrene-D	0.0–0.07%	
	Bicyclogermacrene	0.0–0.15%	
	β-Bisabolene	0.0–0.37%	
	δ-Cadinene	0.0–0.04%	
	Dodecanoic Acid, İsooctylester	0.0–0.06%	
	Camphene	0.0–1.16%	
	β-Myrcene	0.0–5.10%	
	Cis-Ocimene	0.0–7.46%	
	Benzene 1.2-Dimethyl-	0.0–0.06%	
	5-Allyl-4-(1-(P-Aminophenyl)Ethylidenehy	0.0–27.69%	
	Linalool-L	0.0–7.77%	
	Carvacrol Methyl Ether	0.0–0.68%	
	Laurınsaeure,4-Octylester	0.0–0.14%	
	Pulegone	0.0–7.94%	
	Endo-Borneol	0.0–0.25%	
	Lınalool Oxıde Cıs	0.0–0.07%	
	Cyclopentasiloxane, Decamethyl-	0.0–0.02%	
	2,5-cyclohexadiene-1,4-Dione,2-Methyl	0.0–0.13%	
	Dodecane,2,2,11,11-Tetramethyl	0.0–0.03%	
Lupin flakes	Hexanal	237.0 µg/kg	[222]
	(E)-Non-2-enal	16.0 µg/kg	
	γ-Nonalactone	64.9 µg/kg	
	(E,E)-Deca-2,4-dienal	4.3 µg/kg	
	Hexanoic acid	2655.0 µg/kg	
	Pentanoic acid	468.0 µg/kg	
	β-Ionone	23.1 µg/kg	
	Octanal		
	2-Acetyl-1-pyrroline		
	Nonanal		
	2-Isopropyl-3-methoxypyrazine		
	Methional		
	2-Isobutyl-3-methoxypyrazine		
	2-Methylbutanoic acid/3-methylbutanoic acid		
	(E,E)-Non-2,4-dienal		
	(E,Z)-Deca-2,4-dienal		
	3-Hydroxy-2-methyl-pyran-4-on (maltol)		
	trans-4,5-Epoxy-(E)-dec-2-enal		
	δ-Nonalactone		
	3-Hydroxy-4,5-dimethyl-2(5H)-furanone (sotolone)		
	Vanillin		

**Table 12:** Comparative composition of protein, dietary fiber, selected micronutrients, vitamins, and bioactive compounds in seeds of narrow-leaved lupin (*Lupinus angustifolius*) and other economically important grain legumes. Values are presented on a dry matter basis where available. Data are compiled from the published literature.

Component	Narrow-Leaved Lupin (*L. angustifolius*)	Yellow Lupin (*L. luteus*)	White Lupin (*L. albus*)	Soybean (*G. max*)	Pea (*P. sativum*)	Source
Protein (% DM)	32.8–33.9	37.5–40.6	34.7–38.9	35.4–39.9	23.4–24.7	[16]
Dietary fiber (g/kg)	149.0–−159.3	128.3–131.4	112.6–120.6	108.0–114.9	54.4–63.3	[16]
Total carotenoids (mg/kg)	17.20–62.52	6.12–6.33	8.94–12.70	–	–	[67]
Lutein (mg/kg)	up to 35.95	4.30–4.50	6.65–8.77	1.35–32.08	–	[67,68,69]
Phytosterols (mg/kg)	13,603.6–16,007.4	13,434.0–15,655.2	7117.2–11,491.2	–	–	[52]
Calcium (g/kg)	1.59–2.08	–	–	–	0.28–0.35	[117]
Iron (mg/kg)	30.1–31.1	–	–	61.0–99.2	–	[117]
Zinc (mg/kg)	20.8–22.0	46.2–48.1	–	–	–	[117]
Copper (mg/kg)	3.5–3.8	–	–	11.1–14.3	–	[117]
Apigenin heteroside (mg/kg)	665.9–692.0	706.0–1143.7	130.0–465.2	0.68–2.13 *	–	[184,187]
Phenolic acids (mg/kg)	Not detected	10.0–27.6	7.5 **	–	57.5–248.2	[184]
Vitamin B1 (Thiamine) (mg/kg)	5.66–7.12	–	–	9.12	–	[108,157,158]
Vitamin B2 (Riboflavin) (mg/kg)	2.36–2.74	–	–	3.20	–	[108,157,158]

* Total flavonoid content reported for soybean. ** Detected in only one white lupin cultivar. DM—dry matter. “–” indicates that comparative data were not provided in the source reviewed.

**Table 14:** Lupin ingredients used in cosmetics.

Name	Application	Sources
Lupine Amino Acids	Skin care, hair nourishment	[322]
Lupinus albus oil unsaponifiables	Skin care	[323]
Lupinus Albus Protein	Skin care	[324]
Lupinus Albus Seed Extract	Skin care	[325]
Lupinus Albus Seed Oil	Skin care	[326]
Lupinus Luteus Seed Extract	Skin care, adds fragrance	[327]
Lupinus Subcarnosus Symbiosome Extract	Skin care	[328]
Lupinus Texensis Seed Extract	Hair nourishment, nail care, skin care, affects the viscosity of the cosmetic	[329]
Chlorella Vulgaris/Lupinus Albus Protein Ferment	Skin protection and care	[330]
Hydrolyzed Lupine Protein	Antistatic, hair nourishment, skin care	[331]
Hydrolyzed Lupine Protein Octenylsuccinate	Skin protection	[332]
Hydrolyzed Lupine Seed Extract	Hair nourishment, skin care	[333]
Hydrolyzed Lupinus Albus Flower Extract	Skin protection	[334]

**Table 15:** Cosmetics containing lupin products.

Product/Cosmetic	Producer and country	Application as Specified by the Manufacturer	Lupin Ingredient	Source
Bitter Lupine Powder	The Glory of Asia Industrial Co. Jeddah, Saudi Arabia	Skin care	Narrow-leaved lupin seed extract	[335]
Emulsified Body Butter	Lupin Isle Beauty Co. Cavendish, Canada	Skin care	Lupinus Albus (Lupin) Seed Oil	[336]
Koha Lupin Rejuvenating Cream	Koha Queenstown, New Zealand	Skin care	Lupin oil	[337]
green, odmładzanie. (green rejuvenation.)	tołpa Bielenda Group S.A Kraków, Poland	Anti-wrinkle firming cream	Lupin seed extract	[338]
krem do stóp na pękającą skórę pięt (foot cream for cracked heel skin)	ziaja Gdańsk, Poland	Foot cream	Hydrolyzed Lupine Protein	[339]
EKO ONA OLEJEK Z ŁUBINU DO TWARZY (PRZEBARWIENIA)— Lupin oil for the face (discolouration)	Manufaktura kosmetyków EKO ONA Goszczanów, Poland	Brightens and whitens the skin, eliminates freckles and pigment spots	Lupin oil	[340]
OLEJ Z ŁUBINU (lupin oil)	ZróbSobieKrem Prochowice, Poland	Brightens and whitens the skin, eliminates freckles and pigment spots	Lupin oil	[341]
Lupine Protein, Hydrolyzed	Makingcosmetics Redmond, Washington, United States of America	Antistatic, rejuvenating and conditioning properties	Seeds of the lupine plant	[342]
5% Lactic Acid + Lupin Protein and Bakuchiol Extract	Lupin Isle Beauty Co. Cavendish, Canada	Skin care	Lupin Protein	[343]
Luscious Lupin Hand and Body Lotion	Lupin Isle Beauty Co. Cavendish, Canada	Hand and body care	Lupin Seed Oil	[344]
Floral Harmony Facial Moisturizer	Lupin Isle Beauty Co. Cavendish, Canada	Facial care	Lupinus Albus (Lupin) Seed Oil	[345]
Emulsified Body Butter	Lupin Isle Beauty Co. Cavendish, Canada	Body care	Lupinus Albus (Lupin) Seed Oil	[346]
Island Kisses Lip Polish	Lupin Isle Beauty Co. Cavendish, Canada	Lipstick	Lupinus Albus (Lupin) Seed Oil	[347]
Red Mud Scrub	Lupin Isle Beauty Co. Cavendish, Canada	Peeling	Lupinus Albus (Lupin) Seed Oil	[348]
Lupin extract	BEAUTYLAB THE STORE Athens, Greece	Skincare	Lupinus Albus Seed Extract	[349]
Daily Sheer Tinted Mineral Sunscreen Fluid SPF50	Babo Botanicals New York, United States of America	Sunscreen Fluid	Lupinus Albus (White Lupin) Seed Oil	[350]
Hair Loss Lotion	Apivita Markopoulo Mesogaias, Greece	Hair protection	Lupin proteins	[351]
Jo Hansford Everyday Shampoo	Jo Hansford London, United Kingdom	Hair care	Sweet Blue Lupin Peptides	[352]
COLOUR PROTECTION SHAMPOO	My Hair Doctor London, United Kingdom	Hair care	Sweet Blue Lupin Peptides	[353]
REVIIVE SHAMPOO	REVIIVE Newry, United Kingdom	Hair care	Sweet Blue Lupin Peptides	[354]
Ultimate Volume Shampoo	Swell London, United Kingdom	Hair care	Blue Lupin Peptides	[355]
Kuracja wzmacniająca szampon przeciw wypadaniu włosów (strengthening treatment anti-hair loss shampoo)	Ziaja Gdańsk, Poland	Against hair loss	Proteins from white lupin	[356]

**Table 16:** Food products made from lupin and containing lupin.

Product Name	Producer	Product	Lupin Ingredient	Source
VANILLA ICE DREAM	wickedkitchen Minneapolis, Minnesota, United States of America	Ice cream	Lupin protein isolate	[394]
Low Carb Potato Mash	Real Good Food Group Sunshine Coast, Australia	Mashed potato with lupin	Ground Australian lupin	[395]
The Gluten Free Food Co Cup Cake/Muffin Mix	Real Good Food Group Sunshine Coast, Australia	Gluten Free cup cakes	Lupin flakes	[396]
KETO LEMONLICIOUS SLICE BAKE MIX	Real Good Food Group Sunshine Coast, Australia	Slice bake mix	Lupin flour	[397]
Lupreme	Eighthdayfoods Melbourne, Australia	Plant protein	Lupin seeds	[398]
TLC Lupin protein flakes	thelupinco Fremantle, Australia	Flakes	Seeds	[399]
MyEy VollEy	MyEy Düsseldorf, Germany	Egg substitute	Lupin flour	[400]
SALAMI: Rustic Roll	Terra vegane Berlin, Germany	Meat substitute	Lupine flour	[401]
CHORIZO: Mexican Ground				
lupin semolina	Lupins for life Jindera, Australia	Semolina	Lupin seeds	[402]
Toasted Protein Flakes	Lupins for life Jindera, Australia	Flakes	Lupin seeds	[403]
Lupin Crumb	Lupins for life Jindera, Australia	Crumb	Lupin seeds	[404]
Lupin Flour	Lupins for life Jindera, Australia	Flour	Lupin seeds	[405]
Lupinen Protein	Raab Vitalfood Rohrbach an der Ilm, Deutschland	Lupinenprotein Pulver	Seeds	[406]
Wegański SERio Sermeggio	SERio Lublin, Polska	Cheese substitute	Seeds	[407]
SERio Plastry	SERio Lublin, Polska	Cheese substitute	Lupin flour	[408]
Organic Lupin Tempeh with Wild Herbs	tempehmanufaktur Günzach, Germany	Tempeh	Cooked lupins	[409]
Bisque Lupin Tempeh Original	Bisque Tempeh Raleigh, Australia	Tempeh	Lupin beans	[410]
#FINGERFOOD	Saladitos Seville, Spain	Snack	Lupin beans	[411]
#LUPINSLOVERS	Saladitos Seville, Spain	Snack	Lupin beans	[412]
Lulupasta Protein Pasta	wholesomeprovisions Portland, Oregon, United States of America	Lubricating paste	Luin flour	[413]
LUPIN FLOUR	Kaizen United States of America	Flour	Lupin flour	[414]
LOW CARB LUMACHE	Kaizen United States of America	Pasta	Lupin flour	[415]
Cocoa & Dark Choc Chip Lupin Biscuits	Skinnybik Melbourne, Australia	Bar	Lupin flour	[416]
Lufu z Łubinu naturalny	Rumix Prosoya	Tofu	Lupin	[417]
Pates z łubinu klasyczny	Rumix Prosoya	Lubricating paste	lupin	[418]
Marinated lupin	TARWI Rumia, Poland	Snack	Cooked lupins	[419]
Lummus	TARWI Rumia, Poland	Hummus	Cooked lupins	[420]
Essential + Vegan Protein Cacau	TARWI Rumia, Poland	Protein powder	Lupin protein	[421]
Brami lupini	BRAMI Italy	Snack	Lupin seeds	[422]
Lupine White Miso	My Fermentation Paris, France	Miso	Lupin seeds	[423]
Lubinų kava	dvarokavos Kultuvėnai, Lithuania	Coffe	Lupin seeds	[424]
LUPINENWÜRZE Seasoning sauce from lupins	Naturata Marbach am Neckar, Germany	Soya sauce	Lupin seeds	[425]
Date & Butterscotch Lupin Biscuits	SkinnyBik Melbourne, Australia	Cookis	Lupin flour	[426]
Sweet Lupin Beans	Saleh Khalaf -	Seeds to cook	Seeds	[427]
Gluten Free Lupin Loaf	Bodhi’s Bakehouse Fremantle, Australia	Bread	Lupin flour	[428]
Tartinade lupin-butternut	Vivien d’Anjou Soudan, France	Lubricating paste	Seed	[429]
Rotini	Lupii Lupini Bean Powered Bite New York, United States of America	Pasta	Lupin flour	[430]
Almond Butter Cinnamon Raisin	Lupii Lupini Bean Powered Bite New York, United States of America	Butter	Lupin	[431]
Ultra Low Carb Protein Chips—Nacho Cheese	LOKA. Kyneton, Australia	Chips	Lupin Flour	[432]
BIO Keimsprossen-Saat Süßlupine	Kiepenkerl Everswinkel, Germany	Sprouts	Lupin seeds	[433]
Boisson lupin	SOYATOO Tofutown.com Wiesbaum, Germany	Milk/plant-based drink	Lupin seeds	[434]
Lupinenkaffee	kornkreis Giengen an der Brenz (Hürben), Germany	Coffee	Seed	[435]
Lupi RECHARGE	LUPI COFFEE Lille, France	Coffee	Seed	[436]
Craft beer Lupinus with Anterivo coffee Birra artigianale Lupinus al caffè di Anterivo	Birra di Fiemme Cavalese, Italy	Beer	Seeds	[437]
Grappa with toasted lupin from Anterivo	L’Ònes Panchià, Italy	Alcohol	Seed	[438]
Lupin chips	PINARIE Bangkok, Thailand	Chips	Seed	[439]
Lupins roasted	croque lupin Québec, Canada	Snack	Seeds	[440]
Rapunzel Bio Lupinenschrot	Kamelur Legau, Germany	Groats	Seeds	[441]
Bio Lupinen, weiß	Kamelur Legau, Germany	Seeds to cook	Seeds	[442]
Lupinen Flocken	Piowald Mühbrook, Germany	Flakes	Lupin seeds	[443]
BIO LUPINEN|WEISS & SÜSS	süssundclever.de Hofheim am Taunus, Germany	Seeds	Lupin seeds	[444]
SIMPLi REGENERATIVE ORGANIC CERTIFIED^®^ LUPINI BEANS	SIMPLi (eatsimpli) Philadelphia, United States of America	Seed	Seeds	[445]
Lupin Protein Isolate	Lupin Gold Osborne Park, Australia	Protein supplement	Seeds	[446]
Lupinen Vrikadelle	purvegan.de Ramsen, Germany	Meat substitute	Seeds	[447]
Lupinen Rostbratwurst	purvegan.de Ramsen, Germany	Meat substitute	Seeds	[448]
Lupinensauce	purvegan.de Ramsen, Germany	Soya sauce	Seeds	[449]
Organic Lupin Miso	O:CHI Lichtenwörth, Austria	Miso	Seeds	[450]
Lupi- Nature	New Roots AG yellowsunshine.ch Oberdiessbach, Switzerland	Tofu	Seeds	[451]

## Data Availability

No new data were created or analyzed in this study. Data sharing is not applicable to this study.
